# The Facile Synthesis of Exogenous Lewis-Base-Free Amidoalanes: A Structural Comparison

**DOI:** 10.3390/molecules30050986

**Published:** 2025-02-20

**Authors:** Jake Hemsworth, Andrej Vinogradov, William Lewis, Simon Woodward, Darren Willcox

**Affiliations:** 1 Department of Chemistry, University of Manchester, Manchester M13 9PL, UK; 2 School of Chemistry, University of Nottingham, University Park, Nottingham NG7 2RD, UK; 3 Carbon Neutral Laboratories for Sustainable Chemistry, University of Nottingham, Jubilee Campus, Nottingham NG7 2TU, UK

**Keywords:** alane, amido, hydride, synthesis, X-ray

## Abstract

A simple one-pot reaction of LiAlH_4_, AlCl_3_, and a secondary amine HNR_2_ [R = Et, *^i^*Pr, *^i^*Bu, cyclo-C_6_H_11_, (CH_2_)_4_, and (CH_2_)_5_] in hydrocarbon solvents results in the formation of exogenous Lewis-base-free amidoalanes [H_2_Al(NR_2_)]_n_ (n = 2 or 3) as crystalline solids (35–85% yield). In the solid state (seven X-ray structures), all the amidoalanes exist as dimers, with the exception of the pyrrolidine-derived alane which exists as a trimer. As solids, these amidoalanes exhibit significant kinetic stability towards oxygen/moisture allowing the brief (ca. 5 min.) handling of [H_2_Al(N*^i^*Pr_2_)]_2_ in air.

## 1. Introduction

**Figure 1:**
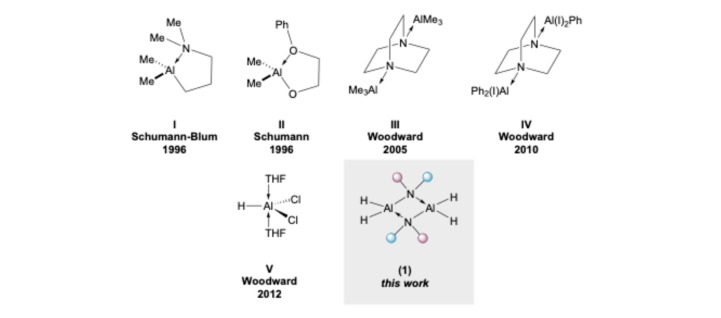
Reported organoaluminium with enhanced air stability [1,2,3,4,5].

The utilisation of organoaluminium reagents in both academia and industry is common practice, especially in reductive chemistries, such as the reduction of carbonyl groups and the metalation of unsaturated C=C/C≡C bonds. Despite their prevalence, these reagents (even ubiquitous LiAlH_4_ to some extent) are frequently pyrophoric in nature, meaning special precautions need to be taken for their safe use. Over the last 30 years, there have been significant efforts made towards stabilizing such air-sensitive compounds, thus making them easier and safer to work with in the laboratory. The first reported examples of air-stabilised aluminium reagents were in 1996, where one of the alkyl groups in R_3_Al was substituted by a group containing a chelating moiety, which imparts stability via the intramolecular formation of a Lewis acid/base pair (Figure 1, **I** and **II**) [1,2].

Since that seminal work, the stabilisation of organoaluminium reagents via Lewis complexation has been the go-to approach to in acquiring user-friendly reagents. In 2005, we popularised the stabilisation of AlMe_3_ using DABCO (DABCO = 1,4-diazabicyclo[2.2.2]octane) to form DABAL-Me_3_ (**III**) [3], which shows remarkable air stability, comparable to LiAlH_4_. This complexation with DABCO was later expanded upon to furnish an equivalent PhAlI_2_ reagent (**IV**) [4]. However, this complex displayed much lower stability in air, and it could only be handled on the bench for short periods of time. Whilst significant progress has been achieved stabilizing alkyl/aryl organoaluminium reagents, the stabilisation of aluminium hydrides has lagged behind. One reason for this is that LiAlH_4_, the most ubiquitous aluminium hydride, already displays reasonable air stability and handling it in air (ca. 5–30 min) does not usually compromise its reactivity, unless aerobic moisture is present [6]. A breakthrough in developing other air-stabilised alanes was reported by us in 2012, where we described simple ethereal Lewis bases, such as THF, as stabilisers of dichloroalane, allowing it to be handled in air without any significant impact on reactivity for 30 min (**V**) [5]. Amidoalanes (**1**) constitute an alternative interesting class of aluminium hydrides as they contain an endogenous Lewis basic moiety. Early studies found that oligomeric species bridged through amido nitrogen atoms are obtained when small substituents are bound to the nitrogen centre. Increasing the steric hinderance enables the isolation of typically dimer species, unless the amine substituents are sterically compact furnishing trimeric species.

The most common routes to species **1** are either the direct reaction of the amine with AlH_3_•NR_3_ (R = Me, Et), the reaction of the amidoaluminium dichloride with LiH, or the generation of AlH_3_ through the *in situ* protonation of LiAlH_4_ via the ammonium salt of the amine [7,8,9,10,11,12,13,14]. Herein, we report a facile, one-pot synthesis of a range of exogenous Lewis-base free amidoalanes [H_2_Al(NR_2_)]_n_ (n = 2–3) and explore the properties of these compounds. Conveniently, multigram quantities of these reagents are easily attained, without the need to use pyrophoric AlH_3_.

## 2. Results and Discussion

### 2.1. Synthesis of Amidoalanes

**Scheme 1:**
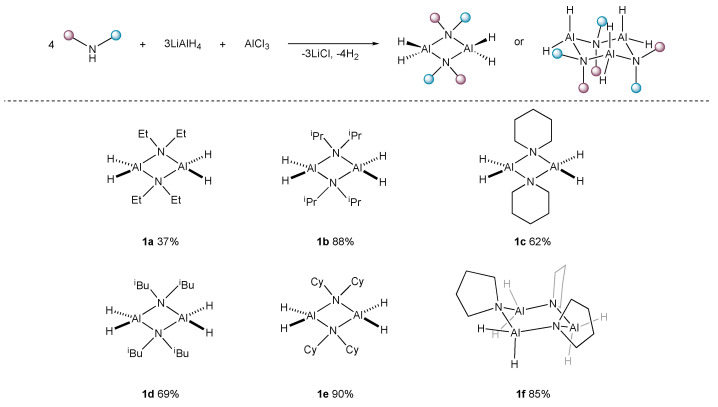
Synthesis of amidoalanes.

Based on our previous work on synthesizing dichloroalane from LiAlH_4_ and AlCl_3_ (1:3 stoichiometry), we reasoned that with appropriate LiAlH_4_ and AlCl_3_ stoichiometries, we could generate ‘AlH_3_’ in situ and we found that this approach would be advantageous as the free volatile primary alane would not be lost from the reaction mixture if it was already datively bound to an amine donor. The choice of reaction solvent mixture for this procedure (Scheme 1) is crucial. The premixing of LiAlH_4_ and AlCl_3_ in petroleum solvents (n-hexane or n-pentane) leads to no reaction due to the insolubility of these species in such solvents. However, upon the slow addition of the *sec*-amine, even at 0 °C, the mixture solubilises and the rapid formation of the amidoalane is evidenced by the evolution of hydrogen gas. This approach completely minimises the formation of free AlH_3_ in the reaction mixture, greatly simplifying the scale-up of the reaction. On completion of the reaction, workup is also facilitated by the use of the hydrocarbon solvent. The only by-product, LiCl, is completely insoluble and is easily separated via cannula filtration under an inert atmosphere, without the need for glovebox procedures. The results from six representative amines are shown in Scheme 1.

This synthetic route is attractive as it can be carried out with small solvent volumes on large scales (up to 20 g is achieved here). Additionally, in most cases, compounds **1a**–**f** can also be prepared using LiAlH_4_, AlCl_3_, and petroleum solvents used directly as supplied, with only the simple (4 Å molecular sieve) drying of secondary amines. This approach typically results in a slight reduction in the isolated yield (10–15%) but does allow the isolation of large quantities of **1** used as a reagent in synthetic organic chemistry, without the requirement for specialist equipment. The structure of **1c** is known in the Cambridge Structural Database (CSD), but no experimental data have been previously published [8]. Since it was reported to be an ‘unstable solid’ decomposing above 0 °C, it is unlikely that **1c** constituted the bulk material in this report [8].

**Scheme 2:**
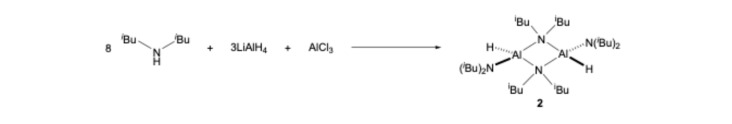
Synthesis of diamidoalane (**2**).

Attempts to extend the chemistry highlighted in Scheme 1 to include chelating diamines, RNHCH_2_CH_2_NHR (R = Me, tBu) were unsuccessful, resulting in only complicated mixtures. However, by changing the stoichiometry of LiAlH_4_:AlCl_3_:HN(*^i^*Bu)_2_ to 3:1:8, we were able to prepare the dimeric monohydrido species [HAl(N*^i^*Bu_2_)_2_]_2_ (**2**) in a 58% yield (Scheme 2). In this case, extended heating (48 h vs. 1–2 h) was necessary to convert intermediate **1d** to **2** in this moderate yield.

### 2.2. Spectroscopic Studies of ***1a**–**f***

The ^1^H NMR spectra of compounds **1a**–**f** in benzene-*d*_6_ clearly show the presence of the amido ligands. The hydride resonance can be identified via very broad resonances in the 4.1–4.6 ppm range (Table 1). All these signals are appreciably broadened due to the associated aluminium quadrupole. The ^27^Al NMR spectra for **1a**–**f** in benzene-*d*_6_ all show a single broad resonance in the 145 ppm region. These resonances fall into the characteristic region for tetrasubstituted aluminium centres with nitrogen ligands. Interestingly, despite being able to observe an Al–H resonance in the ^1^H NMR spectrum for **1e**, we were unable to observe any signals in the ^27^Al NMR spectrum. The ^27^Al NMR spectrum for compound **2** exhibits a singlet which is shifted up-field by ca. 15 ppm compared to the dihydride analogue **1d**, which is characteristic for diamidoalane compounds [9]. It should be noted that whilst the compounds can be isolated cleanly in the solid state, if they are left in solution for prolonged periods of time, degradation and potential ligand scrambling to H_3_Al•N(H)R_2_ can be observed.

In further support of their formulation as alanes, a signal assigned to an Al–H stretch can be detected in every case in the region 1835–1794 cm^−1^ in their IR spectra. The Al–H stretching frequency correlates well with the degree of steric hindrance experienced by the localised Al–H bond.

### 2.3. Single-Crystal X-Ray Crystallographic Studies

**Figure 2:**
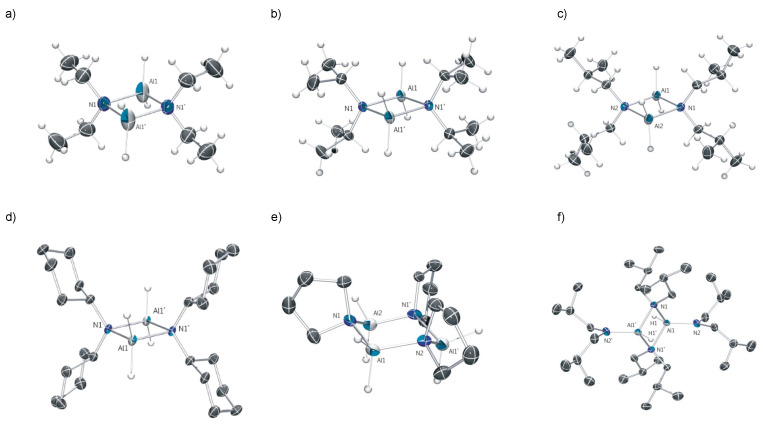
Solid-state structures of amidoalanes: (**a**) **1a** [H_2_Al(NEt_2_)]_2_; (**b**) **1b** [H_2_Al (NPr*^i^*_2_)]_2_; (**c**) **1d** [H_2_Al(NBu*^i^*)]_2_; (**d**) **1e** [H_2_Al (NCy_2_)]_2_; (**e**) **1f** [H_2_Al(NC_4_H_8_)]_3_; (**f**) **2** [HAl(NBu*^i^*_2_)_2_]_2_. Displacement ellipsoids are drawn at the 50% probability level; C-bonded hydrogen atoms are omitted for clarity.

An attractive feature of the amidoalanes **1a**–**e** is their high crystallinity which greatly facilitates their isolation and purification. To confirm their identities, their purities (the complexes are sometimes too reactive for routine CHN combustion analyses) and to determine their degree of aggregation in the solid state, X-ray structural studies on all of the compounds isolated were carried out. The structures of the amidoalanes **1a**–**b**, **1d**–**f**, and **2** are shown in Figure 2. The X-ray structure of **1c** is identical to that crystallographically reported (but without any supporting spectroscopic data) in the literature [8]. Comparative structural data for the dimeric compounds **1a**–**e** and **2** are given in Table 2.

The more sterically encumbered members of the amidoalanes, **1b**, **1d**–**e**, **1f**, and **2**, are all essentially planar. The bridging amido geometries of these dimeric species are all highly similar to one another. The monohydride dimer (**2**) shows the longest Al^…^Al interatomic distance indicative of the greater steric congestion in this compound. This idea is reinforced in the ^1^H NMR spectrum of this compound which shows restricted rotation around the N-CH_2_ bond of the bridging amide (as these appear as separate signals at 22 °C). Remarkably, pyrrolidine-derived **1f** was found to be trimeric in the solid state in our studies. This is in line with expectations for this class of alane derivatives, as sterically compact amido bridges are known to favour higher degrees of oligomerisation [15]. The change in aggregation has little effect on the H–Al–H angle. The values attained in trimeric 1f (ca. 117–118°) being mid-range to that observed in Table 2.

Technical issues aside (difficulties in accurate detection and the small number of examples of this motif in the CSD database), the Al–H distances in this family of molecules deserve comment. Firstly, a wide range of Al-H distances (1.37–1.84 Å) are observed. The average Al–H for Table 2 (1.54 Å) is shorter than pre-existing CSD database averages, both for the most related molecules (1.67 Å) [10] and for all Al–H distances in AlH_2_ motifs (1.66 Å). The observed distances are, in most cases, similar to those in [H_2_Al(N(SiHMe_2_)_2_]_2_ (1.49, 1.52 Å) [11]. In one case, significant differences in the Al–H bond distances within the AlH_2_ unit have been detected (**1d**). Control experiments and checks excluded the following sources for this irregularity: the presence of small amounts of a co-crystallised H_2_Al(μ-N*^i^*Bu_2_)_2_AlHX (X = Cl or other atom) through partial Al–C displacement; a non-representative crystal; the partial conversion of **1d** to **2** in the reaction mixture and its subsequent co-crystallisation; and an insufficiently modelled minor disorder in the structure. At present, we are unable to explain the origin of this effect, and further investigations are continuing in our laboratory in light of this interesting observation.

### 2.4. Air Stability Studies

**Figure 3:**
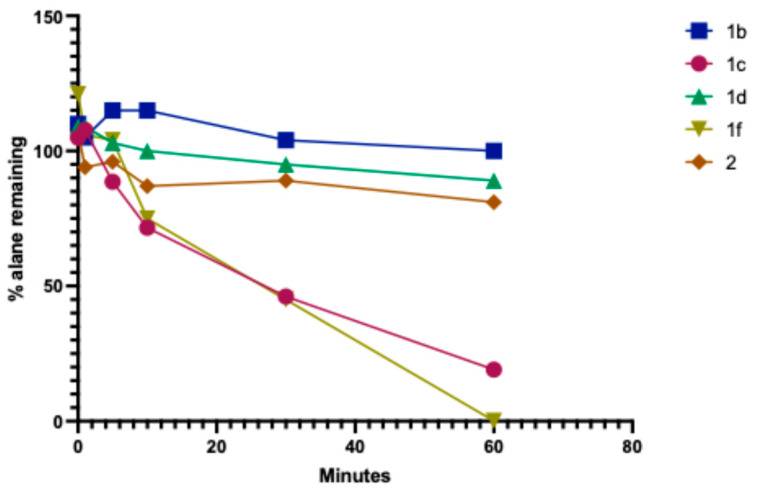
Air stability tests of **1b**, **1c**, **1d**, **1f**, and **2**.

The air stability of compounds **1a**–**f** is improved by the presence of large NR_2_ groups, but these compounds are still rather air-sensitive when in powder forms and should be stored in a glovebox. By preparing the samples in a glovebox, we could attain accurate CHN analyses on **1d** and **1e**. Despite repeated attempts, the other compounds showed significant deviations (~2%) on carbon analyses. Nevertheless, we investigated the use of these species in air as reagents and measured their air stability by way of gas evolution analysis using a gas burette (see ESI). Some of these compounds were proven to be unusable in air; for example, **1c** is rather pyrophoric in the crystalline solid state. Usefully, however, compound **1b** crystallises in larger colourless tablets, and, in this form, it shows some degree of air tolerance (Figure 3). While this is not as good as in DABAL-Me_3_, it does usefully allow for its brief weighing in the open lab [3]. Some (~5%) surface decomposition is evidenced, but the use of **1b** as a potential organic reagent under these conditions is clearly practical, and this is being investigated further.

### 2.5. Reactivity Studies

**Scheme 3:**
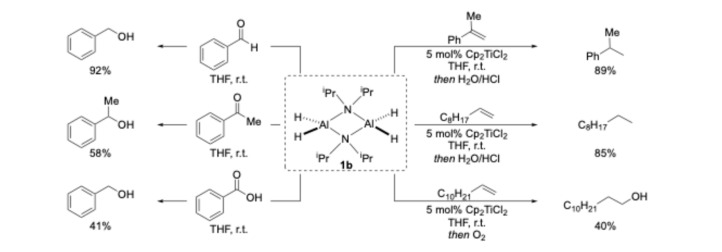
Preliminary reactivity studies using amidoalane **1b**.

Having found that compound **1b** exhibits some air stability, we wanted to test the reactivity of this amidoalane in reduction chemistry (Scheme 3). Compound **1b** readily reduced aldehydes and ketones to the corresponding alcohols in good yields and was also able to reduce benzoic acid to benzyl alcohol when two equivalents of the alane were employed. Our groups have a long-standing interest in transition-metal-catalysed hydroalumination reactions and reasoned that **1b** would be a viable aluminium hydride source for this transformation. To our delight, when **1b** was reacted with a series of olefins under Cp_2_TiCl_2_ catalysis, the corresponding alkanes were furnished in a good-to-excellent yield, upon quenching with aqueous HCl. If the electrophilic quenching reagent is changed from H_2_O/HCl to molecular oxygen, the corresponding alcohol product can be obtained in a moderate yield.

## 3. Materials and Methods

**General**: All procedures were carried out under purified argon in dried solvents (n-hexane and n-pentane dried over LiAlH_4_). Lithium aluminium hydride (Alfa Aesar, 97%, a fine white powder, as supplied) was recrystallised from diethyl ether. For the preparation of diisopropylamidoaluminium dihydride and diisobutylamidoaluminium dihydride, it was found that the same LiAlH_4_ source could be used directly in the preparation, leading to only a slight (10–15%) reduction in the yield compared with recrystallised LiAlH_4_. Aluminium trichloride (Fluka, >99%, cat. number: 06220, pale yellow) was used as purchased. The secondary amines (diethylamine—Alfa Aeser; diisobutylamine, piperidine—Sigma Aldrich, diisopropylamine—Acros, and pyrrolidine—Fluka) were distilled from molecular sieves (4 Å) before use.

**Instrumentation**: ^1^H, ^13^C{^1^H}, and ^27^Al NMR spectra were recorded on a Bruker advance III HD 400 spectrometer (operating frequencies: 400.20 MHz, 128.25 MHz, and 104.27 MHz, respectively). ^1^H and ^13^C{^1^H} NMR chemical shifts were internally referenced to the following residual solvent resonances C_6_D_6_ (benzene-*d*_6_): ^1^H δ = 7.16 ppm and ^13^C{^1^H} δ = 100.64 ppm. NMR samples were prepared under an inert atmosphere in 5 mm J. Youngs NMR tubes. Data were analysed using MestReNova V14.0.0 software. IR spectra were recorded as Nujol mull in KBR disks using a Bruker Tensor 27 spectrometer. The assignment of NMR spectra was based on HMBC, HMQC, ROESY, and DEPT135 techniques as appropriate.

The CCDC deposition numbers are as follows: **1a**—783514; **1b**—783515; **1d**—783516; **1e**—783517; **1f**—783518; and **2**—783519.

Syntheses of dialkylamidoaluminium dihydrides [H_2_Al(NR_2_)]_n_ **1**

Solid LiAlH_4_ (1.00 g, 26.4 mmol) and AlCl_3_ (1.17 g, 8.8 mmol) were suspended in 60 mL of n-hexane or n-pentane. The mixture was cooled to 0 °C using an ice bath. Dry secondary amine (34.0 mmol) was added slowly via a rubber septum using a syringe. During the addition, gas formation (H_2_) was observed. The connection to the Schlenk line vent was kept open in order to avoid a build-up of pressure. After the complete addition of the amine, the rubber septum was replaced (under a flow of argon) by a condenser equipped with a gas bubbler. The mixture was heated at reflux until the gas evolution stopped (typically 1–2 h). After cooling the supernatant, liquids were removed from the solid residue via cannular filtration. The solvent was removed under vacuum to give the crude products. In some cases (**1b** and **1d**), the products began to crystallise upon the partial removal of the petroleum solvents. Alternatively, the crude dialkylamidoaluminium dihydrides were purified by being recrystallised from n-hexane or n-pentane. Remarkably, diethylamidoaluminium dihydride **2** was purified by way of vacuum sublimation (T_sub_ 50–60 °C, 1 mmHg). The preparative scale could be increased without problems to at least 20 g and LiAlH_4_ and AlCl_3_ could be used as supplied with only a small reduction in yield.

[H_2_Al(NEt_2_)]_2_ **1a**. Yield 37%; colourless crystals (blocks); mp. (sealed capillary) 41–42 °C; **^1^H NMR** (400.20 MHz, 298 K, C_6_D_6_): δ/ppm = 0.86 (t, 12 H, CH_3_, ^3^J_HH_ = 7.1 Hz), 2.84 (q, 8 H, CH_2_, ^3^J_HH_ = 7.1 Hz), and 4.13 (br., 4 H, Al-H); **^13^C NMR** (100.64 MHz, 298 K, C_6_D_6_): δ/ppm = 12.3 (CH_3_) and 42.3 (CH_2_); **^27^Al NMR** (104.27 MHz, 298 K, C_6_D_6_): δ/ppm = 150.2; **IR** (NaCl plates, nujol) ν/cm^−1^ = 3446 (vs, br.), 2932 (vs, nujol), 1826 (vs, ν_AlH_), 1453 (vs), 1382 (vs), 1315 (w), 1291 (m), 1261 (w), 1175 (s), 1111 (vs), 1046 (s), 1004 (s), 903 (m), 854 (s), and 732 (s); **MS** (EI, 70 eV): *m*/*z* 201 (38%) (M^+^–H), 187 (13%) (M^+^–CH_3_), 173 (50%) (M^+^–C_2_H_5_), 171 (63%) (M^+^–C_2_H_5_–2H), 157 (77%) (M^+^–CH_3_–C_2_H_5_), and 100 (100%) (monomer–H). ***CARE****!* This compound is somewhat pyrophoric in the solid state.
[H_2_Al(N*^i^*Pr_2_)]_2_ **1b**. Yield 88%; colourless crystals (blocks); mp. (sealed capillary) 133–134 °C; **^1^H NMR** (400.20 MHz, 298 K, C_6_D_6_): δ/ppm = 1.29 (d, 24 H, CH_3_, ^3^J_HH_ = 7.0 Hz), 3.56 (sept., 4 H, CH, ^3^J_HH_ = 7.0 Hz), and 4.30 (br., 4 H, Al-H); **^13^C NMR** (100.64 MHz, 298 K, C_6_D_6_): δ/ppm = 24.7 (CH_3_) and 50.7 (CH); **^27^Al NMR** (104.27 MHz, 298 K, C_6_D_6_): δ/ppm = 143.5; **IR** (NaCl plates, nujol) ν/cm^−1^ = 3585 (m), 3413 (m), 3318 (m), 2925 (vs, nujol), 2600 (m), 2303 (w), 1816 (vs, ν_AlH_), 1592 (m), 1448 (vs), 1390 (vs), 1314 (m), 1261 (w), 1175 (vs), 1126 (vs), 977 (w), 956 (s), 915 (s), and 840 (s); **MS** (EI, 70 eV): *m*/*z* 257 (23%) (M^+^–H), 243 (8%) (M^+^–CH_3_), 227 (20%) (M^+^–2CH_3_–H); 213 (20%) (M^+^–3CH_3_); 129 (6%) (monomer), 128 (100%) (monomer–H), 126 (30%) (monomer–3H), 114 (42%) (monomer–CH_3_), and 86 (57%) (monomer–C(CH_3_)_2_).
[H_2_Al(NC_5_H_10_)]_2_ **1c**. Yield 69%; colourless crystals (blocks); mp. (sealed capillary) 91–92 °C; **^1^H NMR** (400.20 MHz, 298 K, C_6_D_6_): δ/ ppm = 1.11–1.17 (m, 4 H, C4-H_2_), 1.33–1.38 (m, 8 H, C3-H_2_), 2.72–2.75 (m, 8 H, C2-H_2_), and 4.14 (br., 4 H, Al-H); **^13^C NMR** (100.64 MHz, 298 K, C_6_D_6_): δ/ppm = 24.1 (C4), 27.0 (C3), and 51.6 (C2); **^27^Al NMR** (104.27 MHz, 298 K, C_6_D_6_): δ/ppm = 150.3; **IR** (NaCl plates, nujol) ν/cm^−1^ = 3436 (vs, br.), 2923 (vs, nujol), 2499 (m), 2385 (w), 2314 (w), 2257 (w), 2209 (w), 2077 (w), 1990 (w), 1823 (vs, br., ν_AlH_), 1643 (m), 1504 (s), 1452 (vs), 1373 (s), 1312 (m), 1281, (m), 1257 (m), 1190 (s), 1152 (s), 1086 (s), 1030 (vs), 940 (s), 856 (s), and 806 (s); **MS** (EI, 70 eV): *m*/*z* 226 (45%) (M^+^), 225 (52%) (M^+^–H), 224 (24%) (M^+^–2H), 197 (68%); 195 (46%), 113 (8%) (monomer); 112 (50%) (monomer–H), 85 (51%) (piperidine), and 84 (100%) (piperidine–H), 57 (43%), 56 (57%).
[H_2_Al(N*^i^*Bu_2_)]_2_ **1d**. Yield 90%; colourless crystals (blocks); mp. (sealed capillary) 66–67 °C; **^1^H NMR** (400.20 MHz, 298 K, C_6_D_6_): δ/ppm = 0.90 (d, 24 H, CH_3_, ^3^J_HH_ = 6.7 Hz), 1.80–2.00 (m, 4 H, CH), 2.90 (d, 8 H, CH_2_, ^3^J_HH_ = 6.9 Hz), and 4.31 (br., 4 H, Al-H); **^13^C NMR** (100.64 MHz, 298 K, C_6_D_6_): δ/ppm = 22.0 (CH_3_), 27.2 (CH), and 57.0 (CH_2_); **^27^Al NMR** (104.27 MHz, 298 K, C_6_D_6_): δ/ppm = 147.0; **IR** (NaCl plates, nujol) ν/cm^−1^ = 3384 (vs, br.), 2958 (vs, nujol), 1834 (vs, ν_AlH_), 1465 (vs), 1391 (s), 1314 (m), 1268 (m), 1156 (s), 1134 (s), 1082 (vs), 1018 (vs), and 940 (s); **MS** (EI, 70 eV): *m*/*z* 313 (14%) (M^+^–H), 311 (13%) (M^+^–3H), 283 (19%) (M^+^–2CH_3_–H), 271 (23%) (M^+^–C(CH_3_)_2_), 269 (20%) (M^+^–CH(CH_3_)_2_–H), 156 (80%) (monomer –H), 154 (100%) (monomer–3H), and 112 (34) (monomer–CH(CH_3_)_2_–H); **elemental analysis:** anal. calc. for C_16_H_40_Al_2_N_2_: C 61.1, H 12.8, N 8.9, found C 61.4, H 12.5, N 9.0%.
[H_2_Al(NCy_2_)]_2_ **1e**. 85%; colourless crystals (blocks); mp. (sealed capillary) 193 °C (decomposition, yellow); **^1^H NMR** (400.20 MHz, 298 K, C_6_D_6_): δ/ppm = 0.95 (dtt, 4 H, C4-H_ax_, ^2^J_HH_ = 13.0 Hz, ^3^J_HH_ = 13.0, 3.1 Hz), 1.14–1.29 (m, 8 H, C3-H_ax_), 1.47 (app. d, 4 H, C4-H_eq_, ^2^J_HH_ = 13.2 Hz, the small ^3^J_HH_ was not fully resolved), 1.57–1.71 (m, 16 H, C3-H_eq_ overlapped by C2-H_ax_), 2.23 (app. d, 8 H, C2-H_eq_, ^2^J_HH_ = 11.6 Hz, the small ^3^J_HH_ couplings were not fully resolved), 3.21 (dt, 4 H, C1-H_ax_, ^3^J_HH_ = 11.7, 2.6 Hz), and 4.47 (br., 4 H, Al-H); **^13^C NMR** (100.64 MHz, 298 K, C_6_D_6_): δ/ppm = 25.8 (C4), 26.5 (C3), 35.7 (C2), and 61.1 (C1); **IR** (NaCl plates, nujol) ν/cm^−1^ = 3428 (vs, br.), 2924 (vs, nujol), 2664 (s), 1806 (vs, br., ν_AlH_), 1630 (m), 1448 (vs), 1368 (s), 1345 (m), 1259 (s), 1109 (vs), 1025 (vs), 888 (s), and 800 (vs); **MS** (EI, 70 eV): *m*/*z* 417 (10%) (M^+^–H), 387 (13%), 333 (34%) (M^+^–cyclohexyl–H), 208 (100%) (monomer–H), 206 (50%) (monomer–2H), and 138 (50%); **elemental analysis**: anal. calc. for C_24_H_48_Al_2_N_2_: C 68.9, H 11.6, N 6.7, found C 69.3, H 11.4, N 6.8%.
[H_2_Al(NC_4_H_8_)]_3_ **1f**. Yield 62%; colourless crystals (needles); mp. (sealed capillary) 106–107 °C; **^1^H NMR** (400.20 MHz, 298 K, C_6_D_6_): δ/ppm = 1.44–1.69 (m, 12 H, NCH_2_), 2.95–3.40 (m, 12 H, CH_2_), and 4.14 (br., 6 H, Al-H); **^13^C NMR** (100.64 MHz, 298 K, C_6_D_6_): δ/ppm = 25.7 (NCH_2_) and 51.7 (CH_2_); **^27^Al NMR** (104.27 MHz, 298 K, C_6_D_6_): δ/ppm = 144.9; **IR** (NaCl plates, nujol) ν/cm^−1^ = 3515 (vs, br.), 2923 (vs, nujol), 2359 (m), 1794 (vs, br., ν_AlH_), 1456 (vs), 1342 (w), 1291 (w), 1256 (w), 1178 (m), 1102 (s), and 1036 (vs); **MS** (EI, 70 eV): *m*/*z* 297 (5%) (M^+^), 296 (44%) (M^+^–H), 295 (11%) (M^+^–2H), 268 (11%) (M^+^–AlH_2_), 266 (16%) (M^+^–AlH_4_), 264 (10%) (M^+^–AlH_6_), (11%) (M^+^–2H), 219 (37%), 198 (33%) (dimer), 197 (79%) (dimer–H), 196 (28%) (dimer–2H), 169 (68%) (dimer–AlH_2_), 167 (70%) (dimer–AlH_2_), 131 (22%), 99 (8%) (monomer), 98 (100%) (monomer–H), 70 (18%) (pyrrolidine), and 69 (50%) (pyrrolidine–H).

### 3.1. Synthesis of [HAl(N^i^Bu_2_)_2_]_2_ ***2***

All chemicals were used as supplied. Solid LiAlH_4_ (3.0 g, 79.1 mmol) and AlCl_3_ (3.51 g, 26.4 mmol) were suspended in 150 mL of n-hexane. The mixture was cooled to 0 °C using an ice bath. Dry diisobutylamine (36.7 mL, 210.8 mmol) was added slowly via a rubber septum using a syringe. During the addition, gas formation (H_2_) was observed. The connection to the argon line was kept open in order to avoid a build-up of pressure. After complete addition of the amine, the rubber septum was replaced (under a flow of argon) using a condenser equipped with a gas bubbler. The mixture was heated at reflux for 48 h. After cooling, the supernatant liquids were removed from the solid residue by way of cannular filtration. The solvent was removed in a vacuum to produce the crude product. The product began to crystallise upon the partial removal of n-hexane. The crude bis(diisobutylamido)aluminium hydride was purified by being recrystallised from n-hexane.

Yield 17.5 g, 58%; colourless crystals (blocks); mp. (sealed capillary) 107–108 °C; **^1^H NMR** (400.20 MHz, 298 K, C_6_D_6_): δ/ppm = 0.96 (d, 12 H, CH_3_ bridged, ^3^J_HH_ = 6.6 Hz), 1.04 (d, 24 H, CH_3_ term., ^3^J_HH_ = 6.5 Hz), 1.18 (d, 12 H, CH_3_ bridged, ^3^J_HH_ = 6.7 Hz), 2.07 (m, 4 H, CH term.), 2.25 (m, 4 H, CH bridged), 2.97 (d, 8 H, CH_2_ term., ^3^J_HH_ = 6.0 Hz), 3.04 (dd, 4 H, CH_2_ bridged, ^2^J_HH_ = 14.9 Hz, ^3^J_HH_ = 5.6 Hz), 3.12 (dd, 4 H, CH_2_ bridged, ^2^J_HH_ = 13.3 Hz, ^3^J_HH_ = 6.7 Hz), and 4.31 (br., 2 H, Al-H); **^13^C NMR** (100.64 MHz, 298 K, C_6_D_6_): δ/ppm = 21.7 (CH_3_), 22.9 (CH_3_), 23.1 (CH_3_), 27,4 (CH), 27.8 (CH, bridged), 55.7 (CH_2_), and 57.4 (CH_2_, bridged); **^27^Al NMR** (104.27 MHz, 298 K, C_6_D_6_): δ/ppm = 131.1; **IR** (NaCl plates, nujol) ν/cm^−1^ = 3378 (vs, br.), 3175 (vs), 2951 (vs, nujol), 1833 (vs, νAlH), 1464 (vs), 1383 (vs), 1314 (m), 1298 (m), 1262 (s), 1154 (vs), 1102 (vs), 1064 (vs), 1011 (vs), 977 (s) 950 (s), 925 (m), and 862 (s); **MS** (EI, 70 eV): *m*/*z* 526 (13%), 442 (13%), 441 (38%) (M^+^–N*^i^*Bu_2_), 399 (23%), 398 (16%), 314 (45%), 313 (49%), and 154 (100%).

### 3.2. X-Ray Data Collection, Solution, and Refinement

Crystals were mounted in the cold stream of an Oxford Cryosystems open-flow cryostat on a Bruker SMART APEX CCD area detector diffractometer; all data were collected at 150 K. The structures were solved using direct methods and refined by full-matrix least-squares on F2. Crystal and refinement data are summarised in Table 3 and full details are given in the Supplementary Data.

**Hydroalumination of carbonyl compounds:** In a nitrogen-filled glovebox, an oven-dried Schlenk tube was charged with amidoalane **1b** (0.225 mmol), carbonyl compound (0.205 mmol), and THF (1 mL). The flask was removed from the glovebox and stirred for 2 h at room temperature. After 2 h, the reaction was quenched with HCl_(aq)_ and the layers separated, and the aqueous phase was extracted with diethyl ether (3 × 5 mL). The yield was determined by ^1^H NMR spectroscopy using 1,1,2,2-tetrachloroethane as the internal standard.
**Hydroalumination of olefins:** In a nitrogen-filled glovebox, an oven-dried Schlenk tube was charged with amidoalane **1b** (0.225 mmol), olefin (0.205 mmol), Cp_2_TiCl_2_ (2.55 mg, 0.01 mmol), and THF (1 mL). The flask was removed from the glovebox and stirred for 3 h at room temperature. After 3 h, the reaction was either quenched with HCl_(aq)_ or O_2_-bubbled through the reaction and then quenched with HCl_(aq)_. The layers were separated, and the aqueous phase was extracted with diethyl ether (3 × 5 mL). The yield was determined by ^1^H NMR spectroscopy using 1,1,2,2-tetrachloroethane as the internal standard.

## 4. Conclusions

The direct reaction of LiAlH_4_, AlCl_3_, and secondary amines constitutes a highly effective and economical route to dialkylamidoalanes. The isolation and handling of these materials are facilitated by their high crystallinity. This preparative route offers advantages over traditional routes to these compounds using H_3_Al•NR_3_ adducts that have led, in some cases in the past, to reports of partially or even erroneously characterised materials. Some of these hydrides have great potential to be used as reagents in organic chemistry due to their high solubility and, in some cases (**1b**), usable levels of air stability. Compound **1b** was found to reduce a range of carbonyl containing substrates and undergo a titanium-catalysed olefin hydroalumination.

## Figures and Tables

**Table 1:** Selected spectroscopic data for compounds **1a**–**f** and **2**.

Compound	Et (1a)	*^i^*Pr (1b)	-(CH_2_)_5_- (1c)	*^i^*Bu (1d)	Cy (1e)	-(CH_2_)_4_- (1f)	*^i^*Bu (2)
^1^H NMR (δ_Al-H_) (ppm)	4.13	4.30	4.14	4.31	4.47	4.14	4.31
^27^Al NMR (ppm)	150.2	143.5	150.3	147.0	-	144.9	131.1
IR (ν_Al-H_) (cm^–1^)	1826	1816	1794	1823	1834	1807	1833

**Table 2:** Comparison of selected amidoalane structures **1a**–**f** and **2** ^a^.

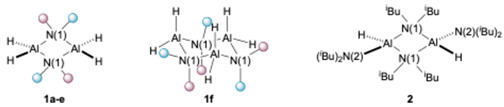								
Structural Parameter	Et (1a)	*^i^*Pr (1b)	-(CH_2_)_5_- (1c) ^a^	*^i^*Bu (1d)	Cy (1e)	-(CH_2_)_4_- (1f)	*^i^*Bu (2)	CSD Ave. (*N* = 17) ^b^
Al—N(1)/Å	1.946(2)	1.9631(12) 1.9640(12)	1.94	1.964(2) 1.968(2) 1.970(2) 1.971(2)	1.961(3) 1.978(3)	1.862(2) 1.9343(5) 1.9354(5) 1.991(2)	1.975(3) 1.991(3)	1.95
Al—N(2)/Å	-	-	-	-	-	-	1.820(3)	1.89
Al—H/Å	1.56(4) 1.60(4)	1.46(2) 1.46(2)	1.75	1.42(3) 1.47(3) 1.78(3) 1.84(3)	1.48(4) 1.55(3)	1.490(8) 1.497(11) 1.520(7) 1.520(10)	1.43(3)	1.67
Al··Al/Å	2.756(2)	2.7499(9)	2.76	2.7442(10)	2.759(2)	3.2442(3) 3.2452(3) 3.2452(3)	2.8197(19)	2.84
R–N(1) ^c^/Å	1.488(2) 1.492(3)	1.515(2) 1.517(2)	1.49	1.491(3) 1.493(3) 1.496(3) 1.498(3)	1.508(4) 1.509(4)	1.5203(8) 1.5298(7)	1.497(4) 1.498(4)	1.50
N(1)–Al–N(1)/^o^	89.86(8)	91.10(4)	89.5	91.48(8) 91.72(8)	91.07(10)	108.48(3) 107.17(3) 110.84(3)	89.39(11)	84.9
Al–N(1)–Al/^o^	90.14(8)	88.90(4)	90.5	88.47(8) 88.33(8)	88.93(10)	113.99(3) 114.64(3)	90.61(11)	93.5
N(2)–Al–H/^o^	-	-	-	-	-	-	116.7(13)	117.5
R–N(1)–R/^o^	111.9(2)	119.38(10)	109.1	112.2(2) 112.5(2)	120.4(2)	105.19(4) 105.99(6)	113.5	110.1
H–Al–H/^o^	102.1(17)	122.3(10)	121	113.2(14) 118.0(14)	122(2)	116.7(4) 118.6(5)	-	108.3

^a^ CSD structure QAGNAO. ^b^ Average of all 17 structures containing H_2_Al(μ-NY_2_)AlH_2_ fragments in the CSD database (Y = any substituent). ^c^ Measured at the α-C atom.

**Table 3:** Crystal and refinement data for **1a**, **1b**, **1d**–**1f** and **2**.

Compound	1a	1b	1d	1e	1f	2
formula	C_8_H_24_Al_2_N_2_	C_12_H_32_Al_2_N_2_	C_16_H_40_Al_2_N_2_	C_24_H_48_Al_2_N_2_	C_12_H_30_Al_3_N_3_	C_32_H_74_Al_2_N_4_
Fw	202.25	258.36	314.46	418.60	297.33	568.91
crystal system	monoclinic	monoclinic	orthorhombic	triclinic	monoclinic	monoclinic
space group	*P*2_1_/*c*	*C*2/*c*	*P*2_1_2_1_2_1_	*P*ī	*P*2_1_/*m*	*P*2_1_/*n*
wavelength [Å]	0.71073	0.71073	0.71073	0.71073	0.71073	0.71073
temperature/K	150(2)	150(2)	150(2)	150(2)	150(2)	150(2)
*a*, Å	7.351(1)	14.514(3)	9.1662(7)	6.613(1)	6.332(1)	10.377(2)
*b*, Å	12.859(1)	15.439(3)	14.3798(10)	12.546(1)	15.113(1)	8.685(1)
*c*, Å	7.219(1)	8.661(2)	15.8971(11)	15.162(1)	9.274(1)	21.34(1)
*α*, deg	90	90	90	98.28(1)	90	90
*β*, deg	90.27(1)	122.23(1)	90	92.08(1)	103.33(1)	99.29(1)
*γ*, deg	90	90	90	90.12(1)	90	90
*V*, Å^3^	682.3(1)	1641.8(6)	2095.4(3)	1244.0(1)	863.6(1)	1898.1(5)
Z	2	4	4	2	2	2
crystal dimensions/	0.10	0.17	0.40	0.12	0.19	0.04
mm	0.30	0.27	0.49	0.19	0.44	0.18
	0.40	0.57	0.63	0.32	0.50	0.20
crystal shape	block	tablet	block	tablet	tablet	plate
*ρ*_calc_, Mg/m^3^	0.984	1.045	0.997	1.118	1.143	0.995
*Θ* range/°	2.77–25.04	2.12–27.48	2.56–25.1	2.28–27.26	2.26–27.53	1.93–25.05
*μ*_abs_ [mm^−1^]	0.177	0.160	0.023	0.129	0.209	0.100
*F*(000) [e]	224	576	704	464	324	640
index ranges	−8 < *h* < 8	−13 < *h* < 18	−10 < *h* < 10	−8 < *h* < 8	−7 < *h* < 7	−12 < *h* < 12
	−15 < *k* < 15	−19 < *k* < 20	−15 < *k* < 17	−16 < *k* < 16	−17 < *k* < 17	−10 < *k* < 10
	0 < *l* < 8	−11 < *l* < 9	−16 < *l* < 18	−19 < *l* < 19	−11 < *l* < 11	−24 < *l* < 25
no. meas. refl.	6599	5118	10,851	14,659	9008	13,559
no. indep. reflns	1215	1861	3707	5591	1587	3372
*R*_int_	0.032	0.022	0.023	0.081	0.019	0.062
no. reflns. observed	1027	1578	3424	3894	1502	2700
parameter/restraints	64/0	85/0	202/0	269/0	116/0	184/0
GooF (*F*^2^)	1.06	1.09	1.05	1.12	1.08	1.09
*R* [*I* > 2σ(*I*)]/*wR*_2_	0.048/0.125	0.061/0.169	0.0466/0.128	0.085/0.171	0.030/0.085	0.079/0.163
*R*_1_ (all)/*wR*_2_	0.060/0.137	0.067/0.176	0.0497/0.131	0.118/0.184	0.031/0.087	0.101/0.172
*ρ*_rest_ (max/min) [e·nm^−3^]	582/−131	1038/−212	1020/−160	502/−348	263/−142	414/−270

## Data Availability

Supporting spectral data are found in the Supporting Information.

## Supplementary Materials

The following supporting information which includes the set-up for the air-stability and the ^1^H, ^13^C and ^27^Al NMR spectral images can be downloaded at the following link: https://www.mdpi.com/article/10.3390/molecules30050986/s1.
