# Supplementary material for: The Facile Synthesis of Exogenous Lewis-Base-Free Amidoalanes: A Structural Comparison
Source: Molecules. 2025 Feb 20;30(5):986. doi: 10.3390/molecules30050986 (PMC11901829; doi:10.3390/molecules30050986)
Supplement: Supplementary file 1 [file molecules-30-00986-s001.zip › molecules-3476354-supplementary.pdf]

Facile synthesis of Exogenous Lewis base free amidoalanes: a structural comparison

Jake Hemsworth [1,a], Andrej Vinogradov [2,a], William Lewis [2] Simon Woodward [3,*] and Darren Willcox [1,*]

[1] Department of Chemistry, University of Manchester, Manchester, M13 9PL, United Kingdom.
[2] School of Chemistry, University of Nottingham, University Park, Nottingham NG7 2RD, United Kingdom.
[3] Carbon Neutral Laboratories for Sustainable Chemistry, University of Nottingham, Jubilee Campus, Nottingham NG7 2TU, United Kingdom.
[a] equal contribution
* Correspondence: SW: simon.woodward@nottingham.ac.uk and DW: darren.willcox@manchester.ac.uk

Table of Contents

Air-stability data. 3

Spectral images of amidoalanes 4

1. Air-stability data

The air-stability of the aluminium hydrides was determined using a gas burette according to the Sigma Aldrich Technical Bulletin AL-123.[1] The gas burette is assembled as illustrated below.

Procedure: In an argon filled glovebox, a Schlenk tube was charged with the corresponding aluminium hydride (100 mg) and attached to the gas burette. The tap of the Schlenk tube was opened and the levelling bulb was adjusted to give a zero reading for the level of distilled water in the burette. A 1M solution of aqueous hydrochloric acid (1 mL) is added slowly through the septa on the Schlenk tube using a needle. Hydrolysis of the amidoalanes is extremely rapid, being complete in a few seconds. The levelling bulb was again adjusted so that a final reading could be obtained for the amount of H_2 gas generated.

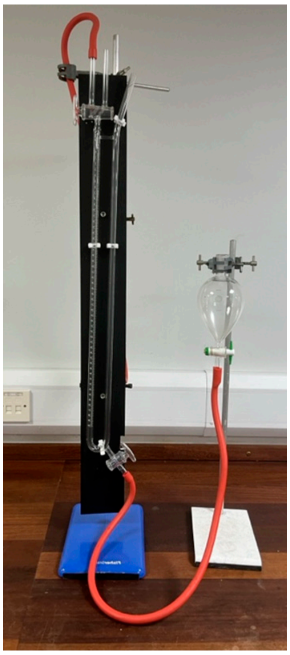

Time	% Al-H remaining				
(min)	**1b**	**1c**	**1d**	**1f**	**2**
0	110	104.9	109	121	107
1	105	107.8	109	106	94
5	115	88.6	103	104	96
10	115	71.6	100	75	87
30	104	46.1	95	45	89
60	100	19.1	89	0.1	81

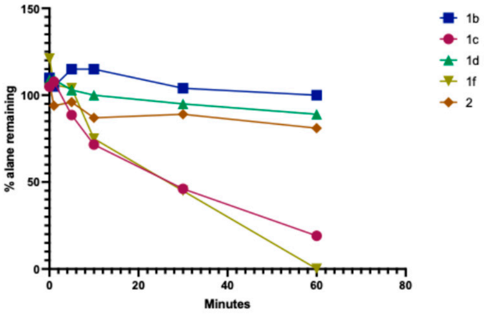

[1] https://www.sigmaaldrich.com/deepweb/assets/sigmaaldrich/product/documents/371/557/al_techbull_al123.pdf

2. Spectral images of amidoalanes

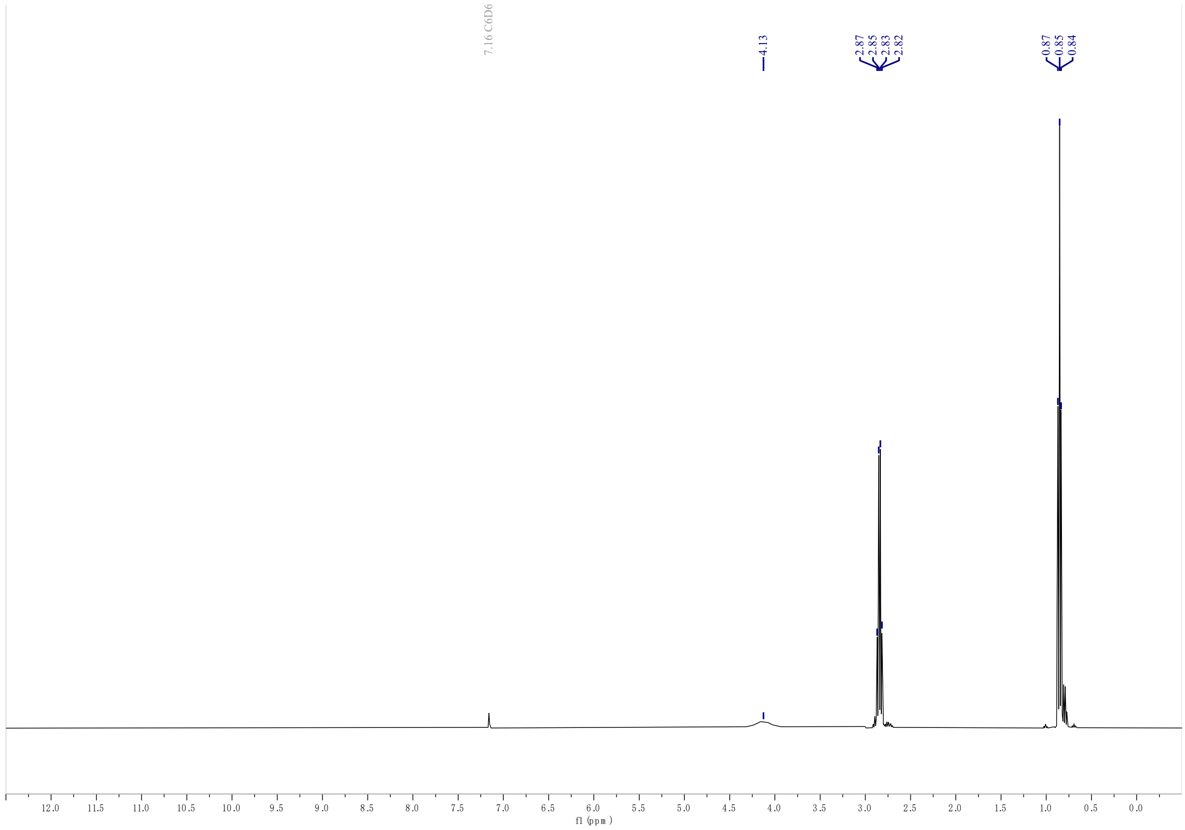

Figure S1. ^{1}H NMR (400.13 MHz, C_6D_6) of **1a**

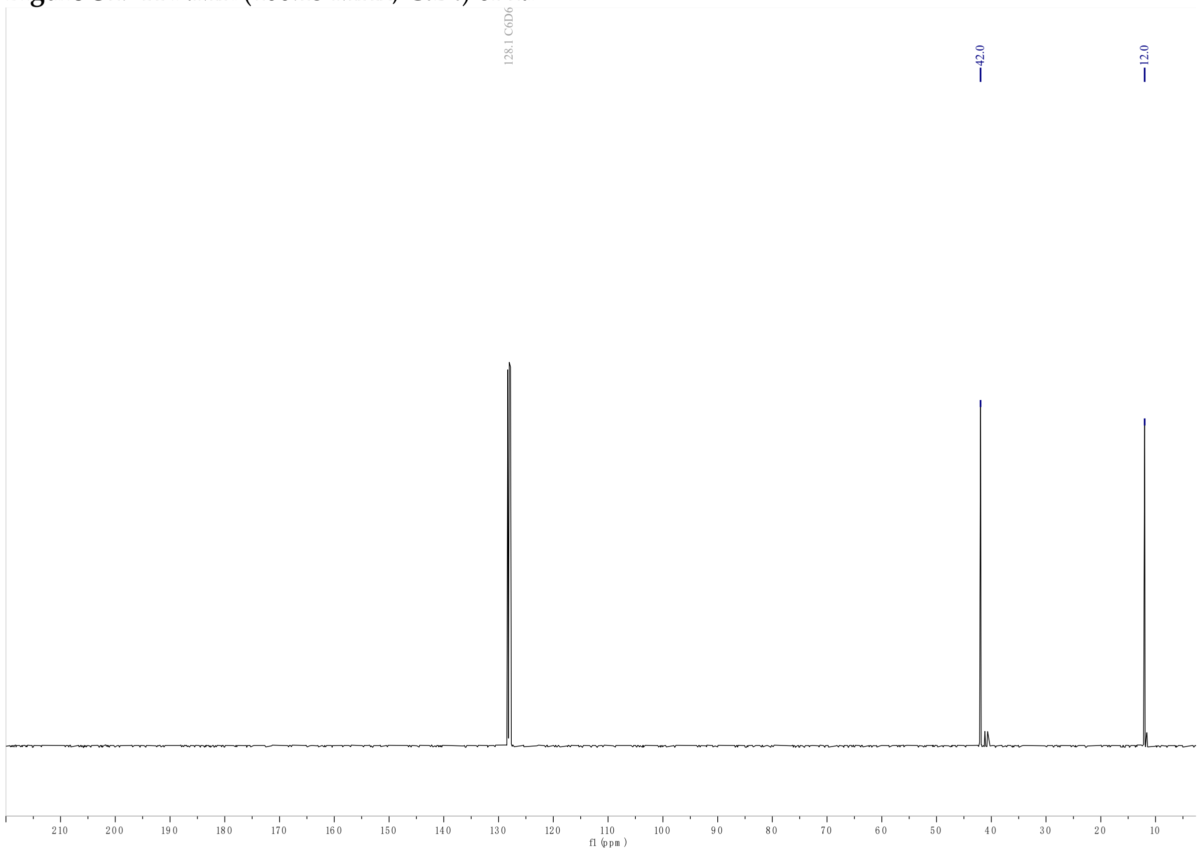

Figure S2. ^{13}C NMR (100.64 MHz, C_6D_6) of **1a**

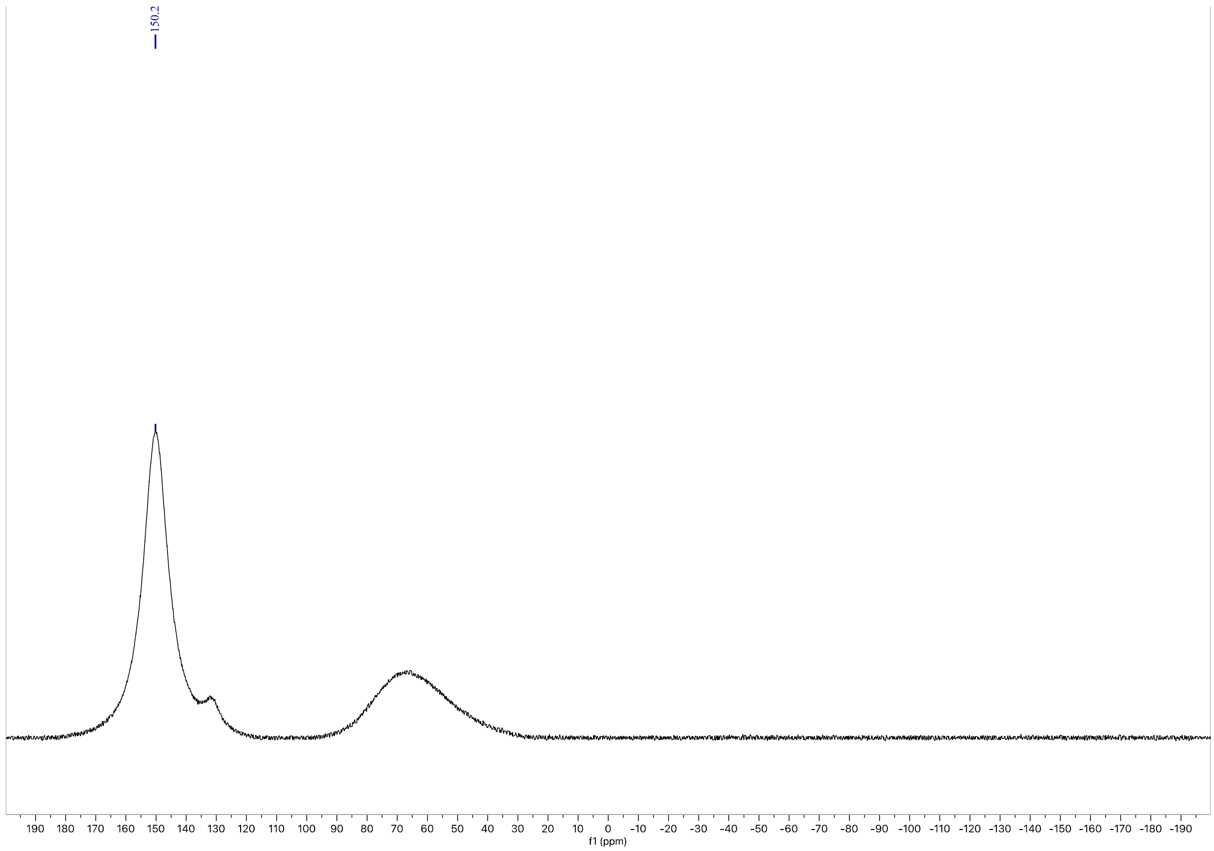

Figure S3. ^{27}Al NMR (104.27 MHz, C_6D_6) of **1a**

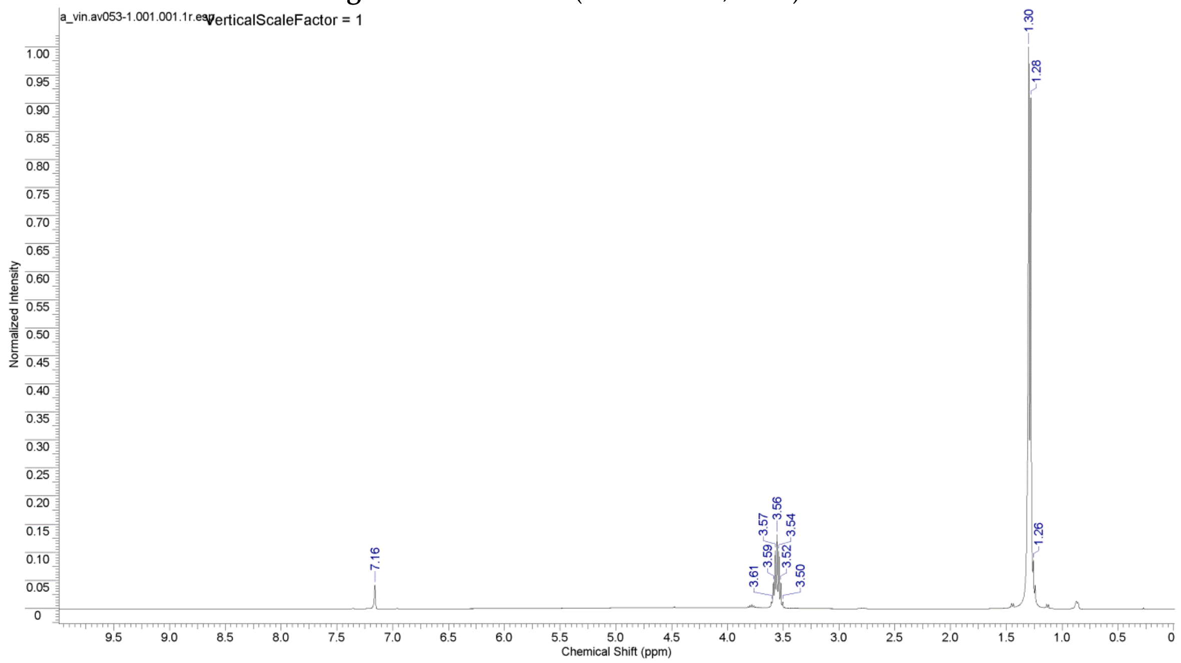

Figure S4. ^{1}H NMR (400.20 MHz, C_6D_6) of **1b**

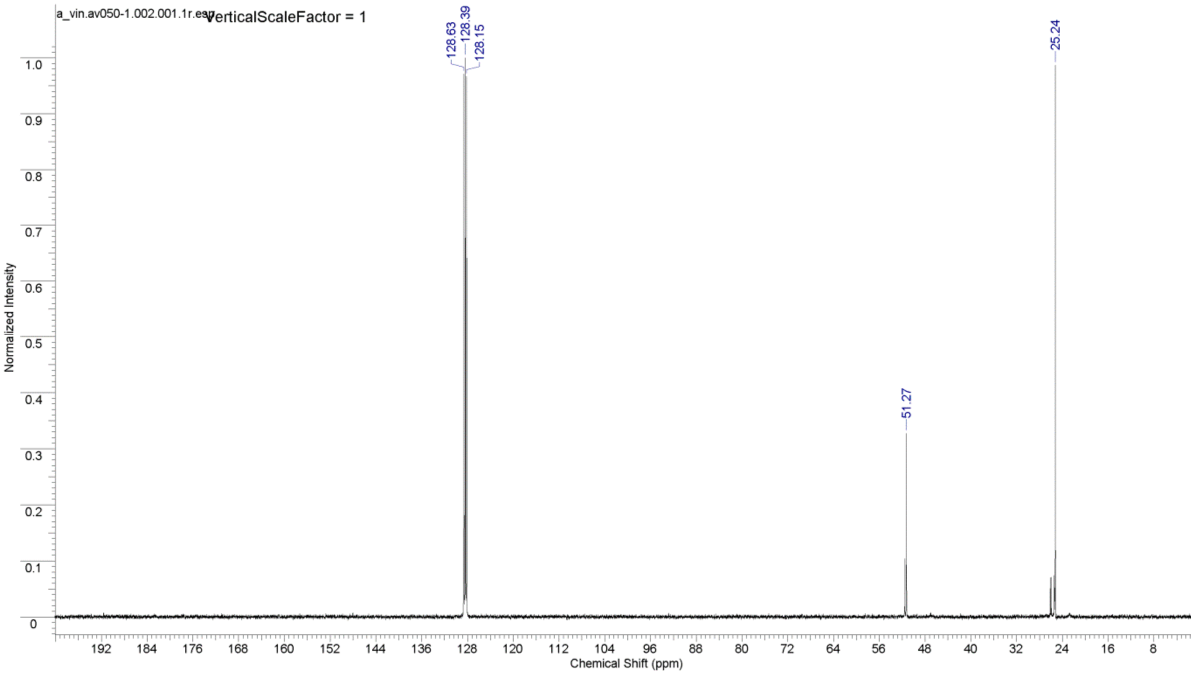

Figure S5. ^{13}C NMR (100.64 MHz, C_6D_6) of **1b**

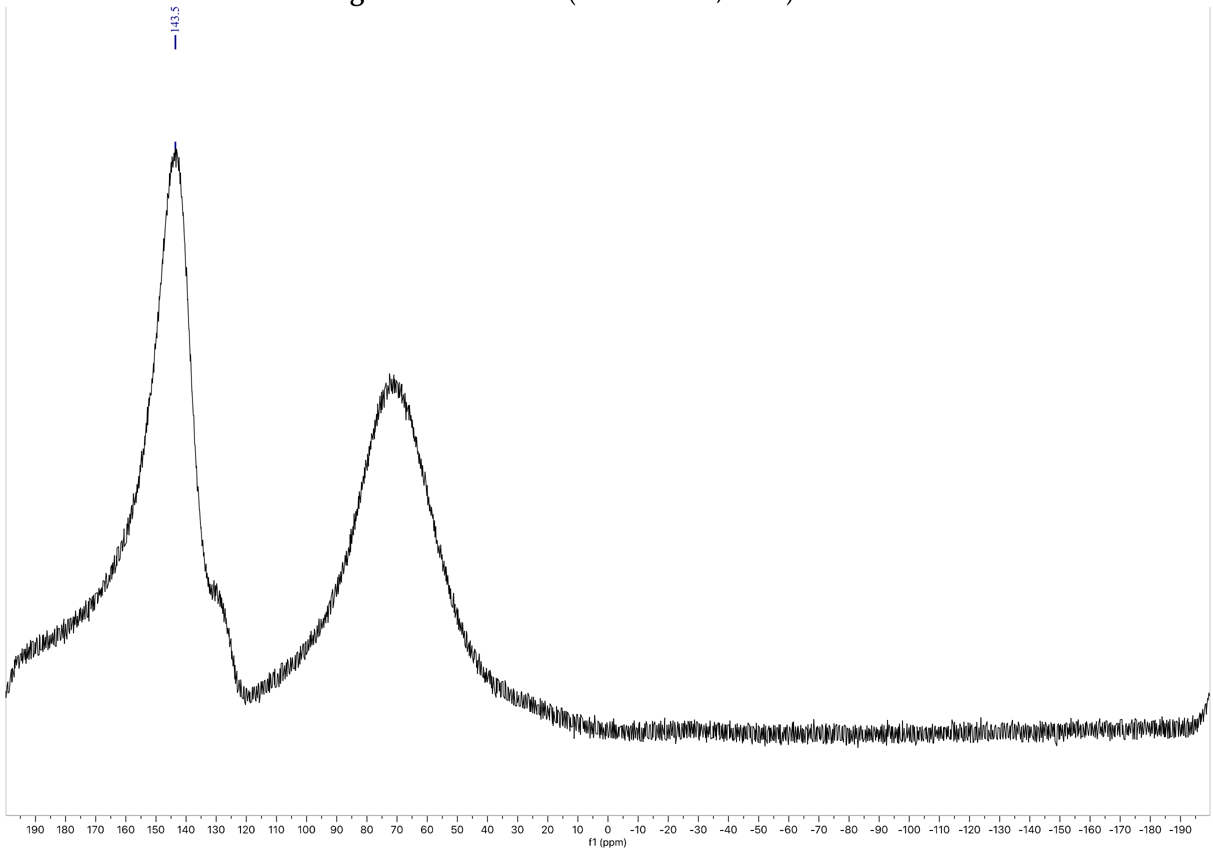

Figure S6. ^{27}Al NMR (104.27 MHz, C_6D_6) of **1b**

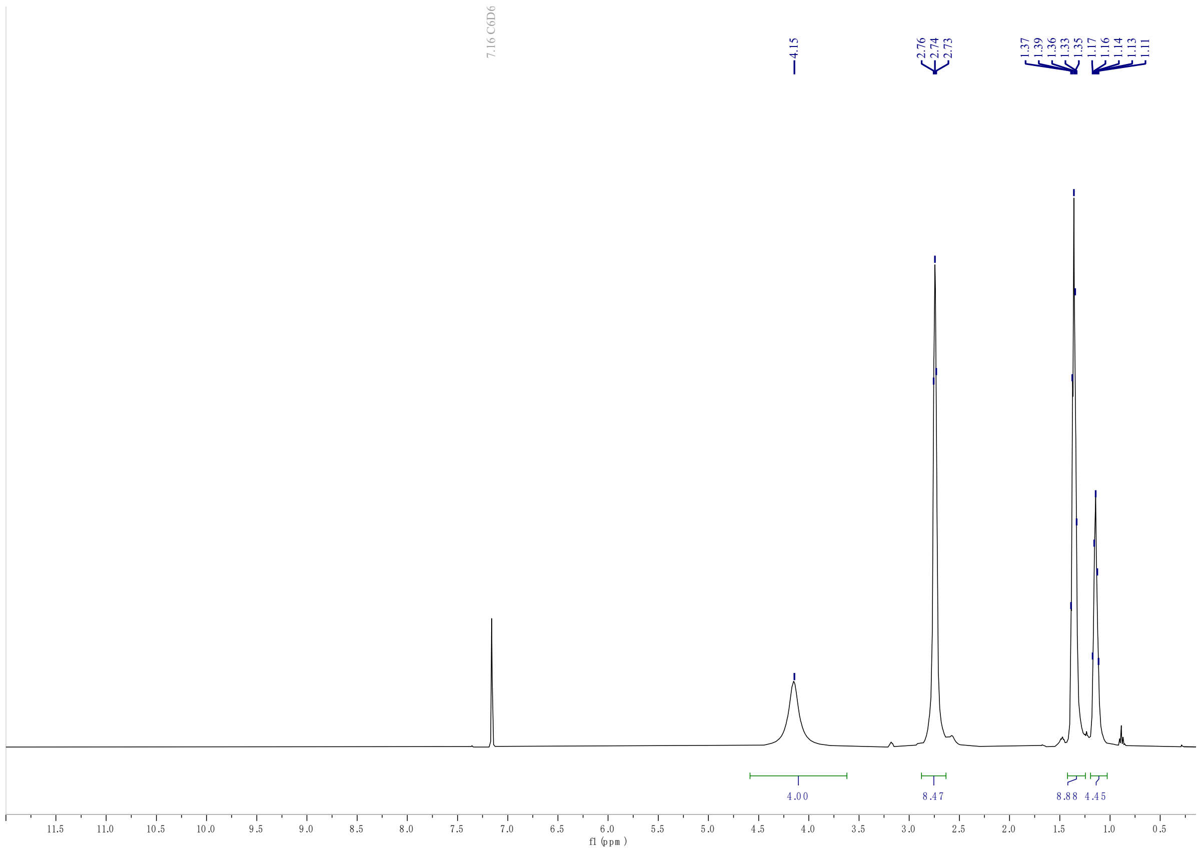

Figure S7. ^{1}H NMR (400.20 MHz, C_6D_6) of **1c**

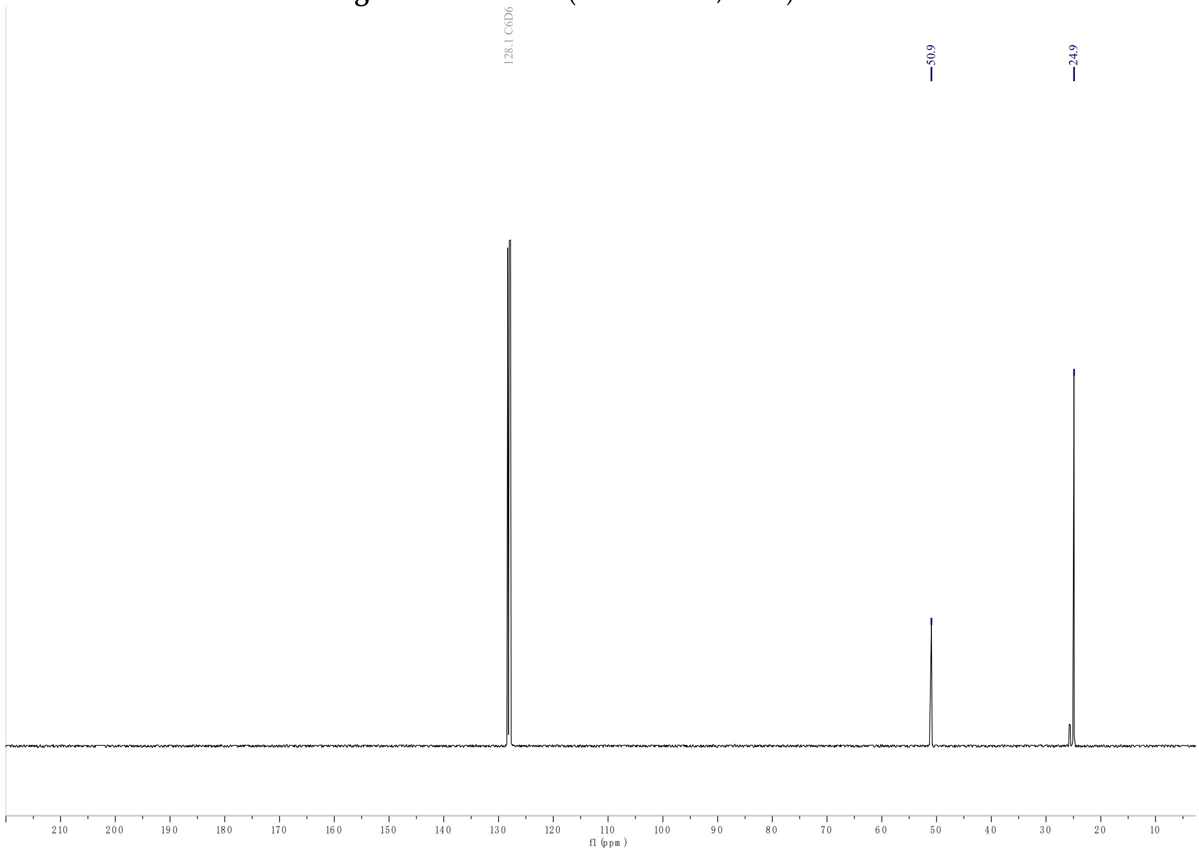

Figure S8. ^{13}C NMR (100.64 MHz, C_6D_6) of **1c**

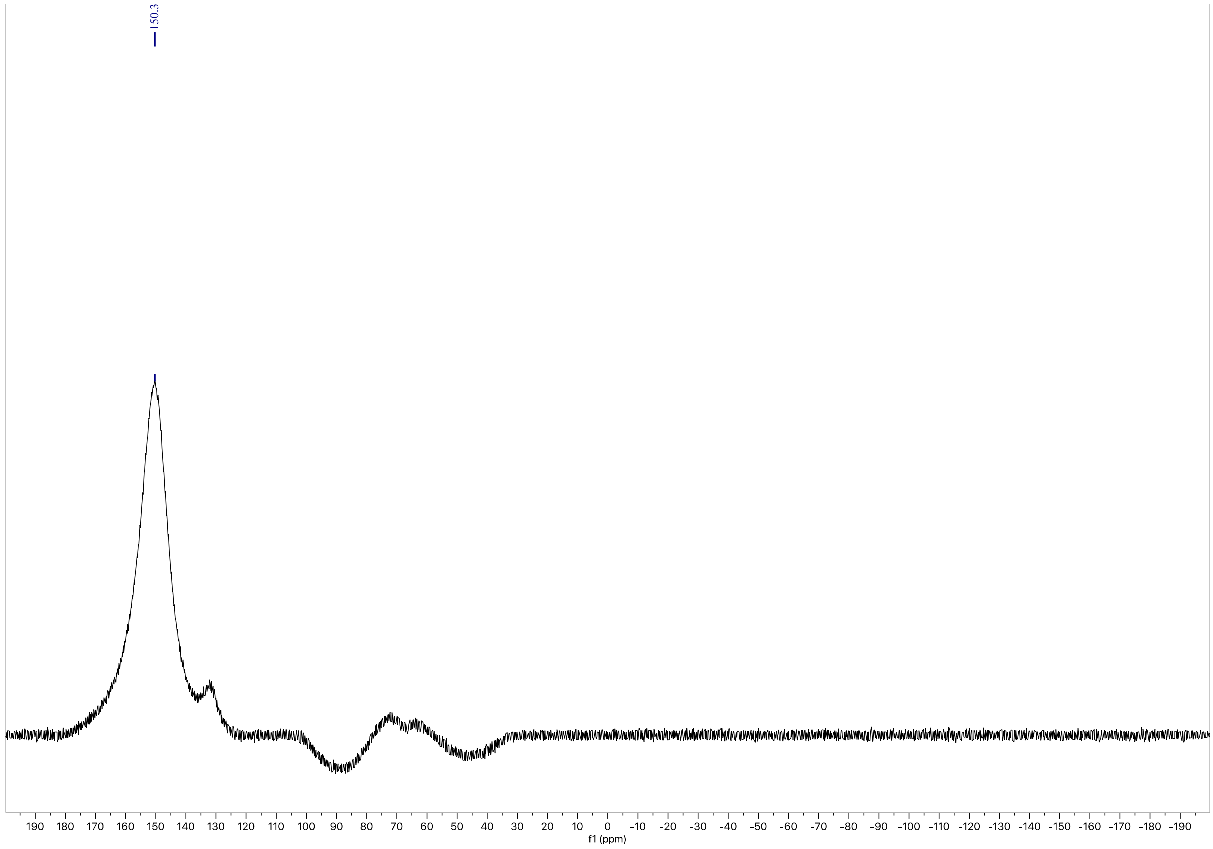

Figure S9. ^{27}Al NMR (104.27 MHz, C_6D_6) of **1c**

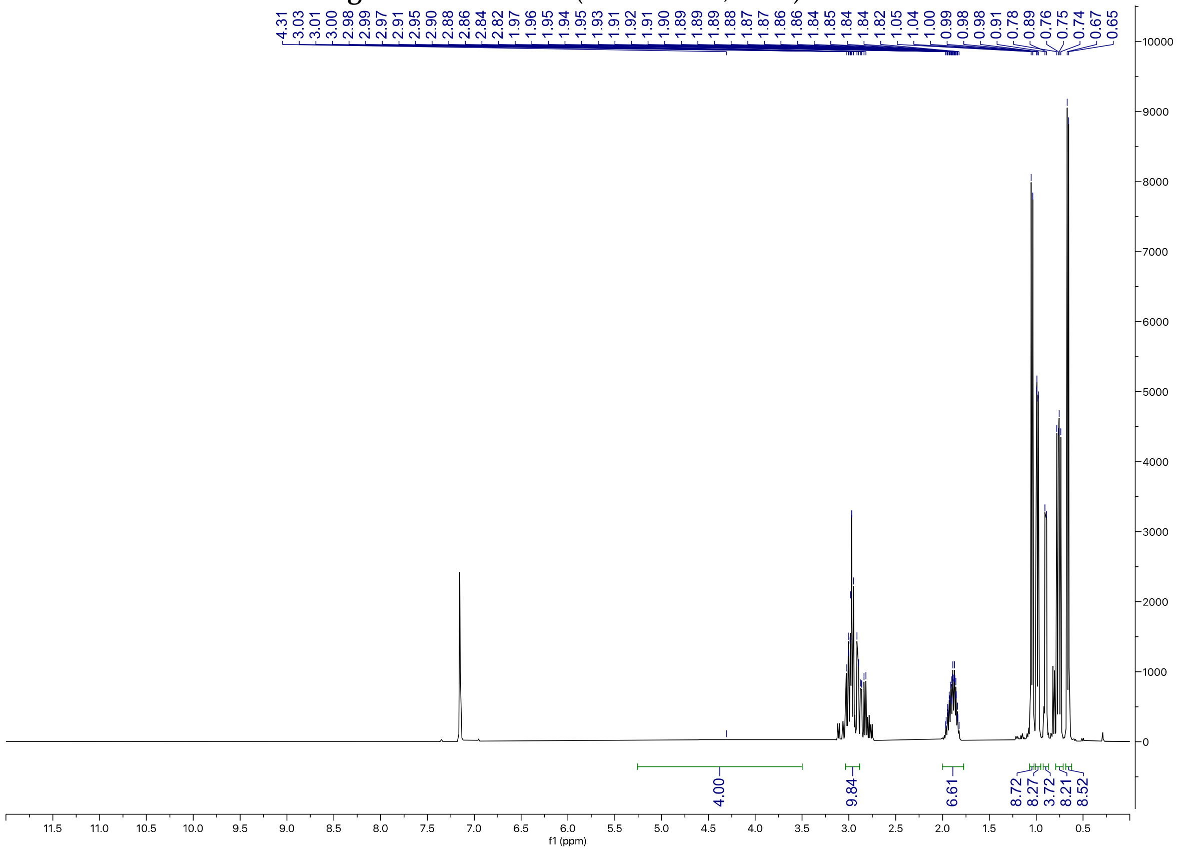

Figure S10. ^{1}H NMR (400.20 MHz, C_6D_6) of **1d**

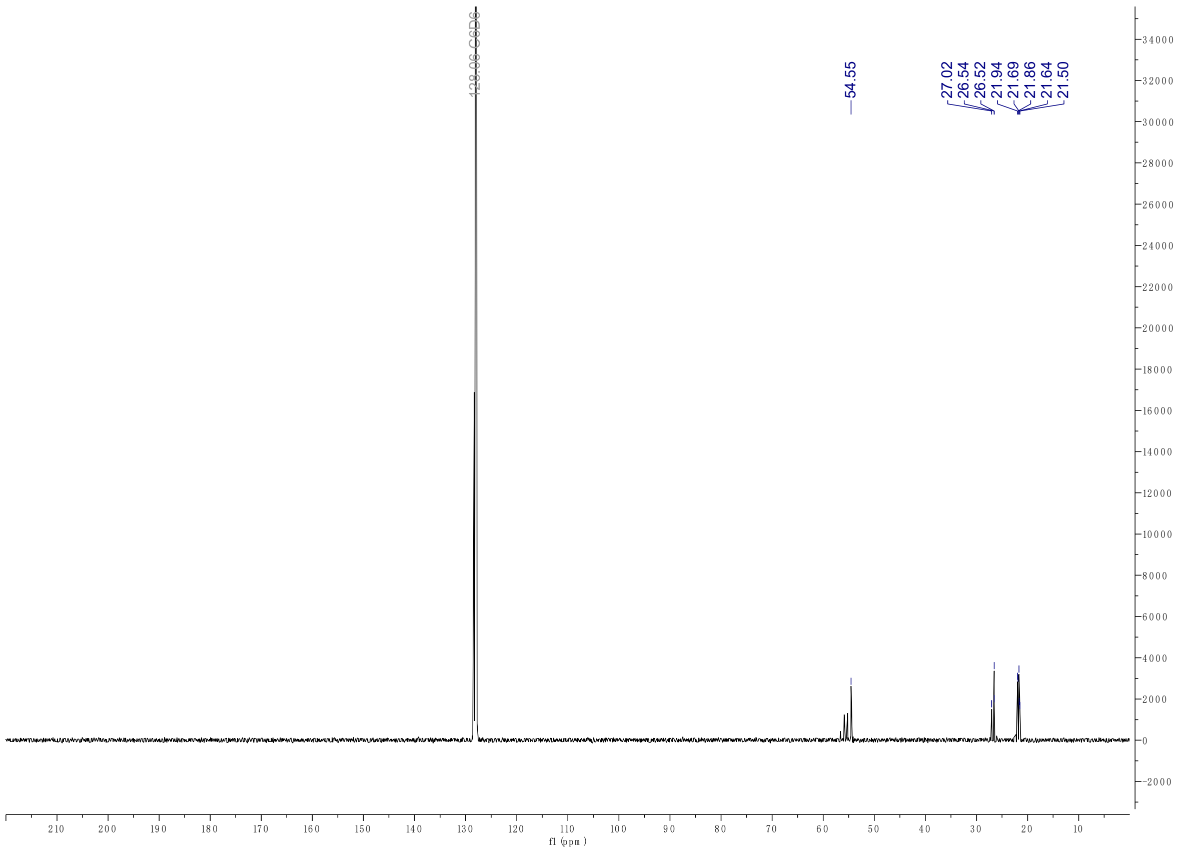

Figure S11. ^{13}C NMR (100.64 MHz, C_6D_6) of **1d**

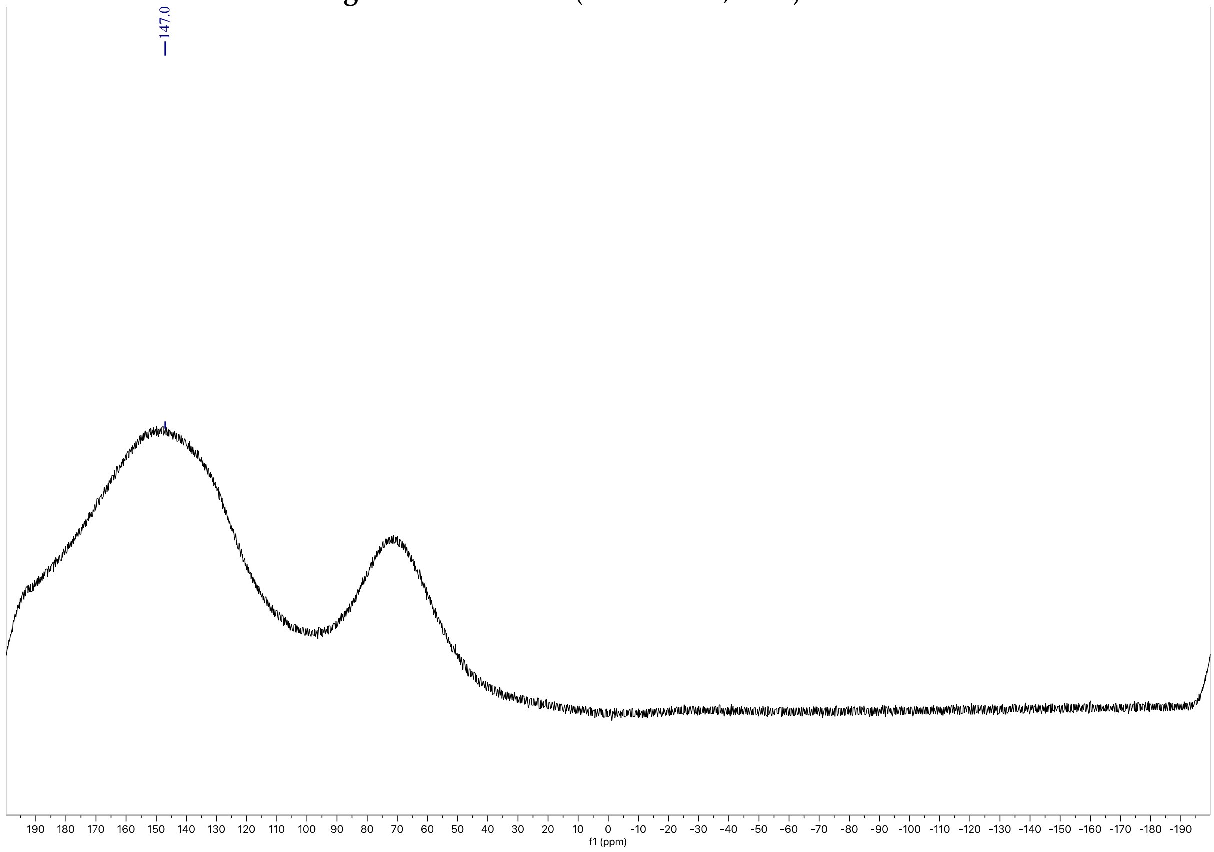

Figure S12. ^{27}Al NMR (104.27 MHz, C_6D_6) of **1d**

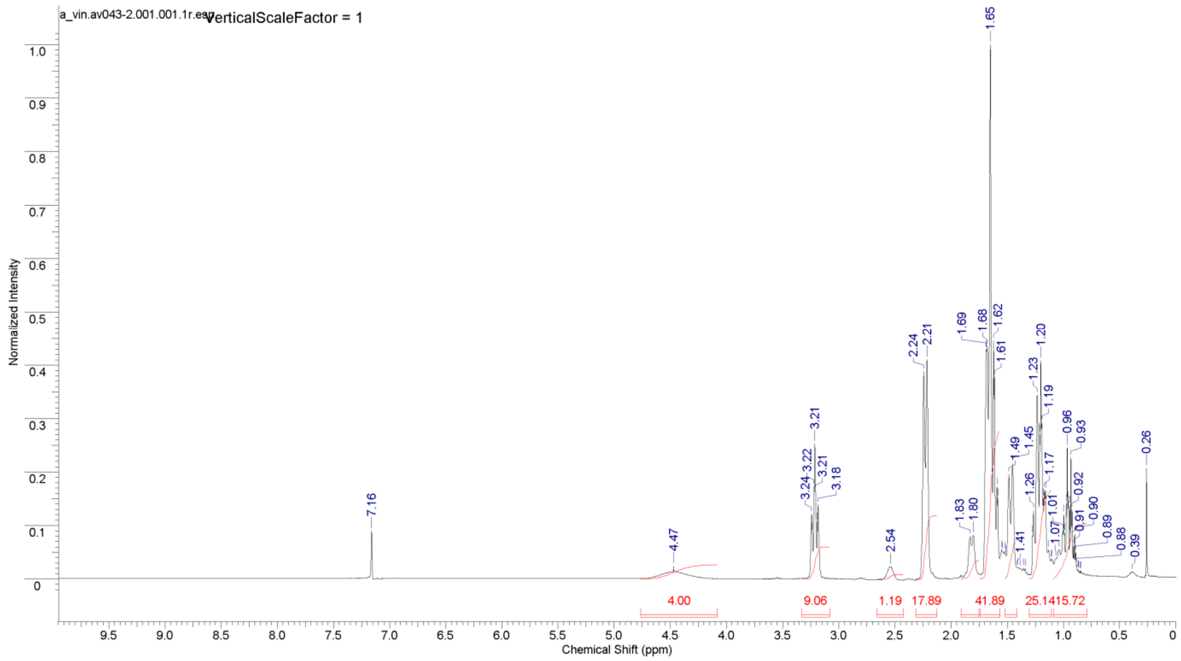

Figure S13. ^{1}H NMR (400.20 MHz, C_6D_6) of **1e**

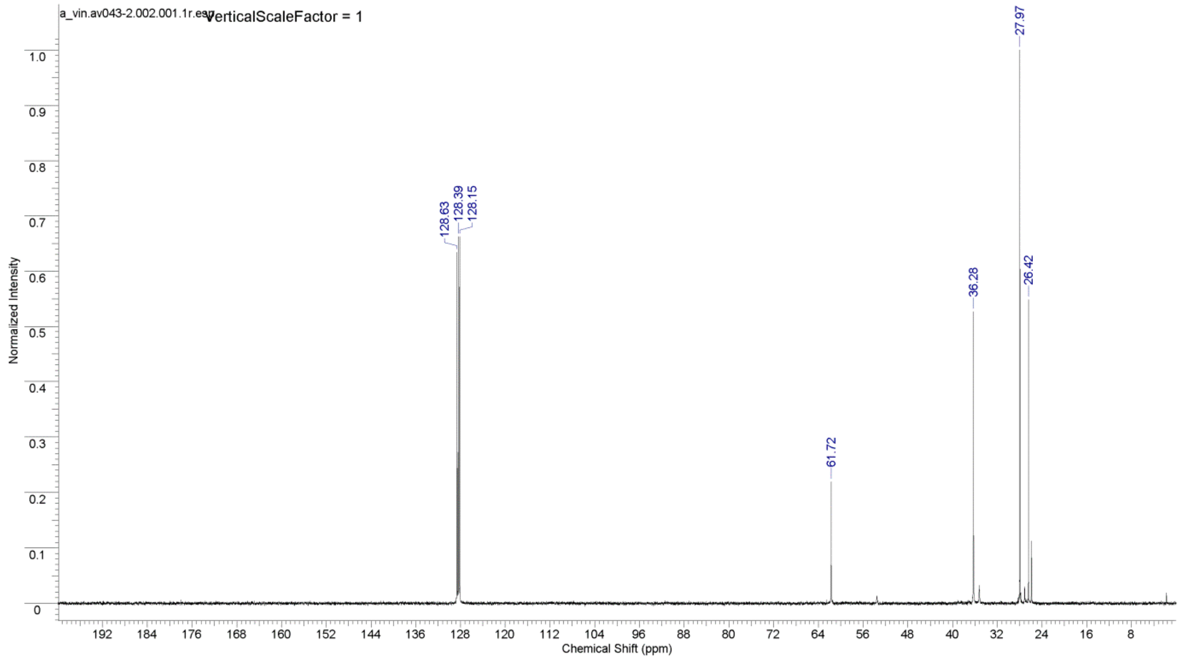

Figure S14. ^{13}C NMR (100.64 MHz, C_6D_6) of **1e**

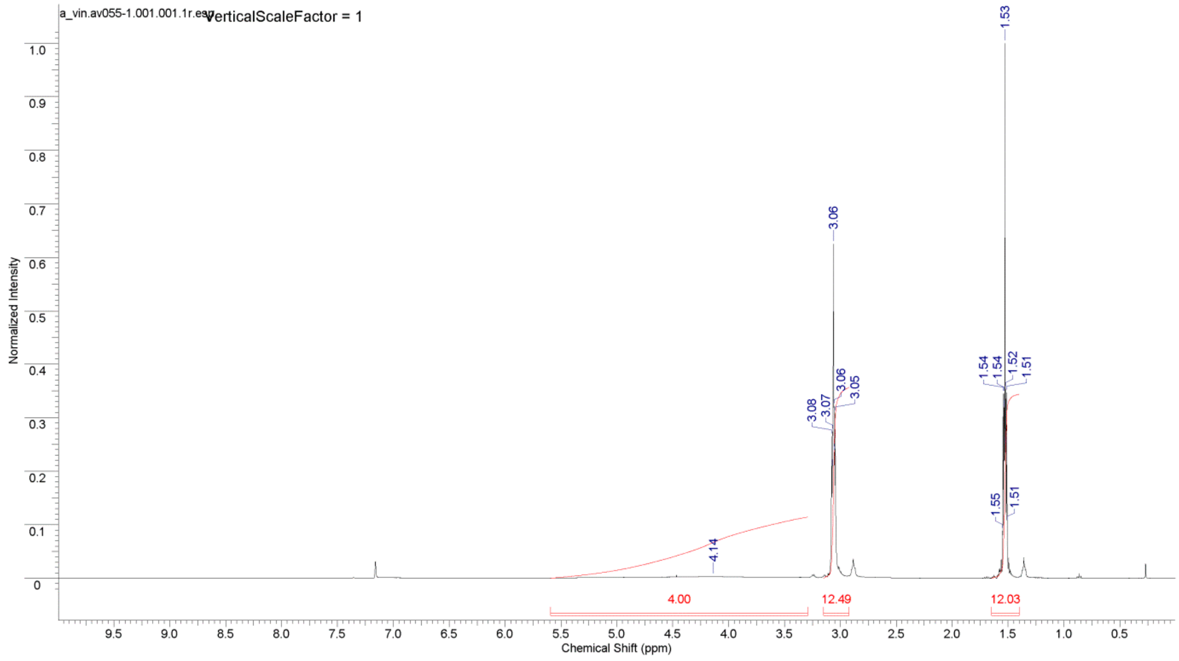

Figure S15. ^{1}H NMR (400.20 MHz, C_6D_6) of **1f**

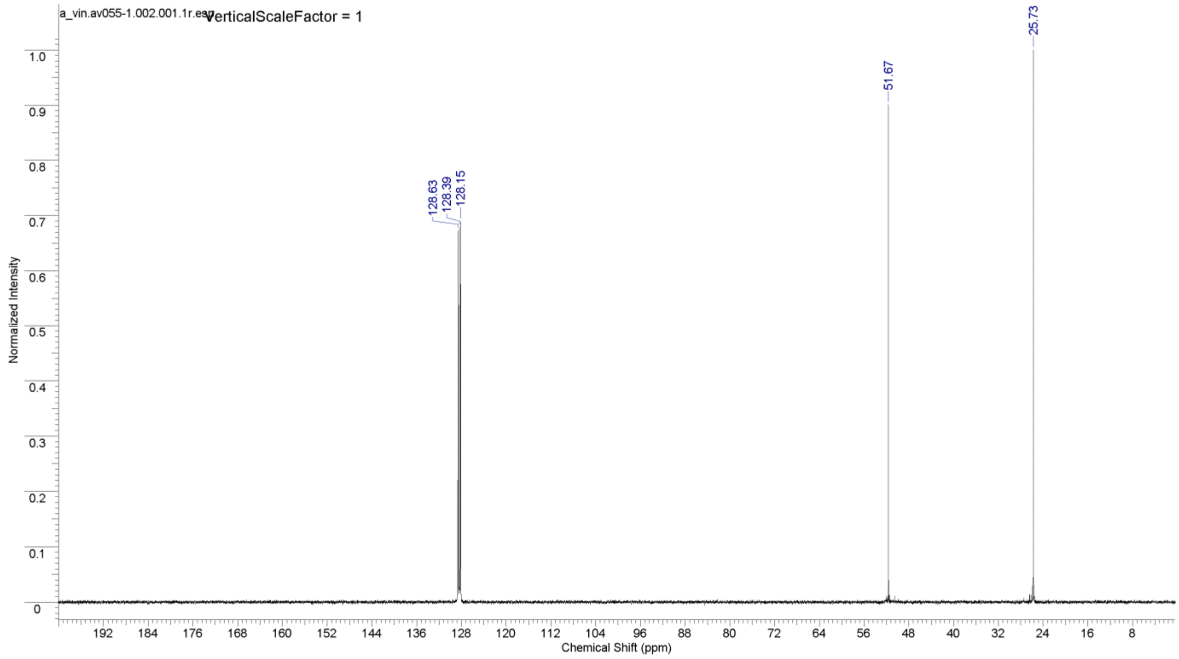

Figure S15. ^{13}C NMR (100.64 MHz, C_6D_6) of **1f**

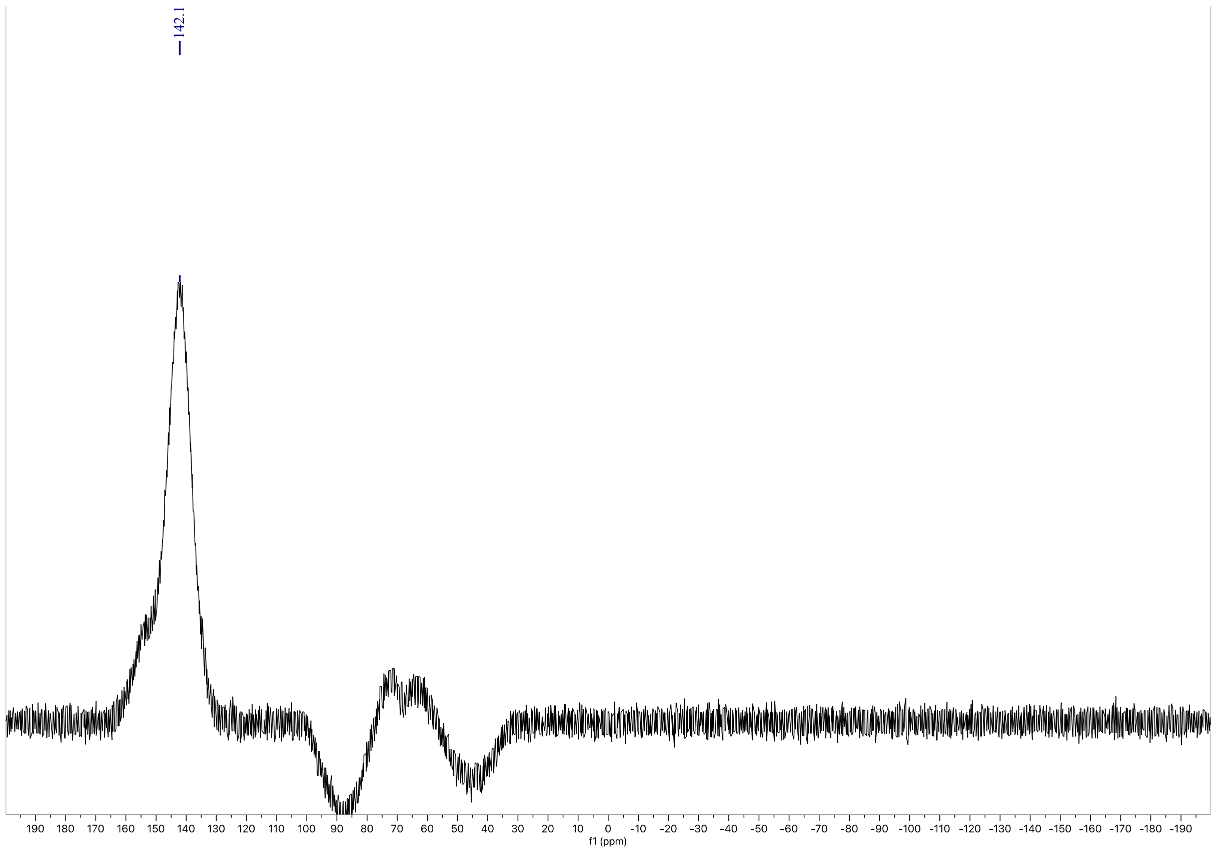

Figure S16. ^{27}Al NMR (104.27 MHz, C_6D_6) of **1f**

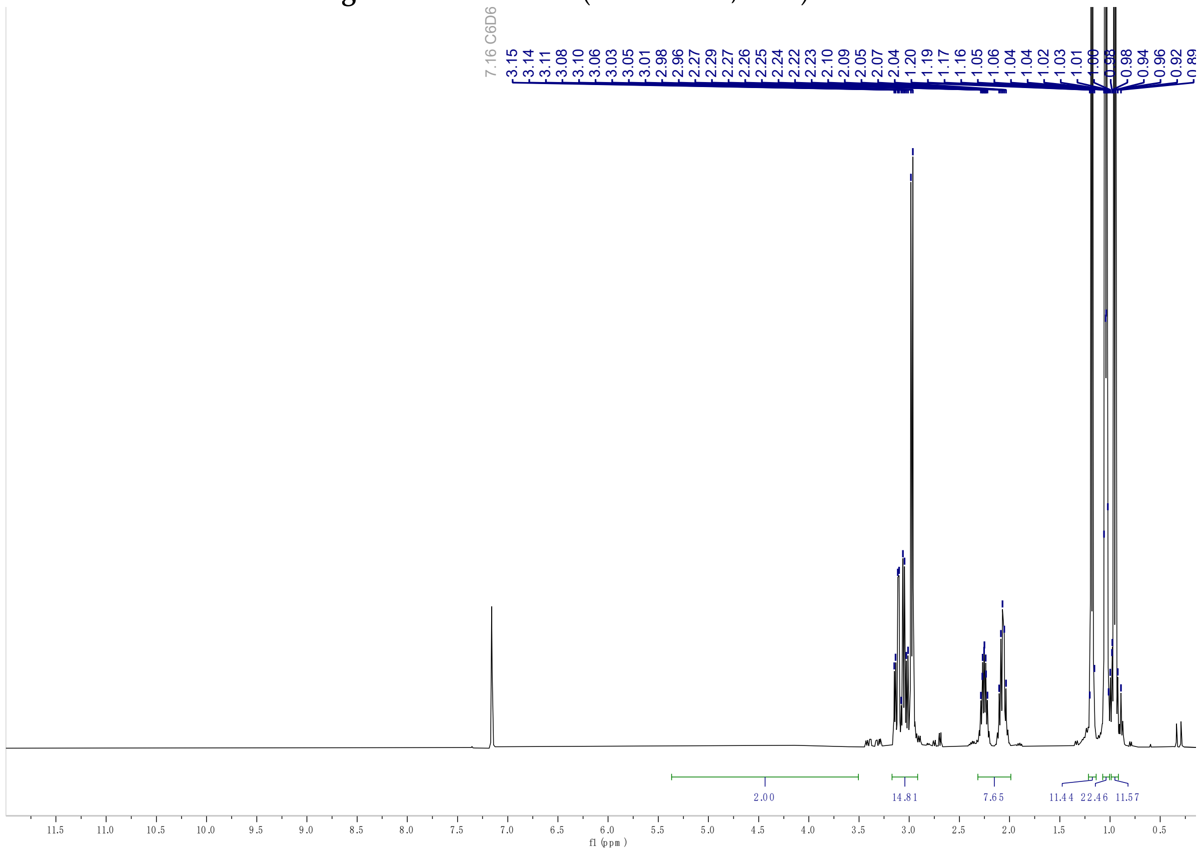

Figure S17. ^{1}H NMR (400.20 MHz, C_6D_6) of **2**

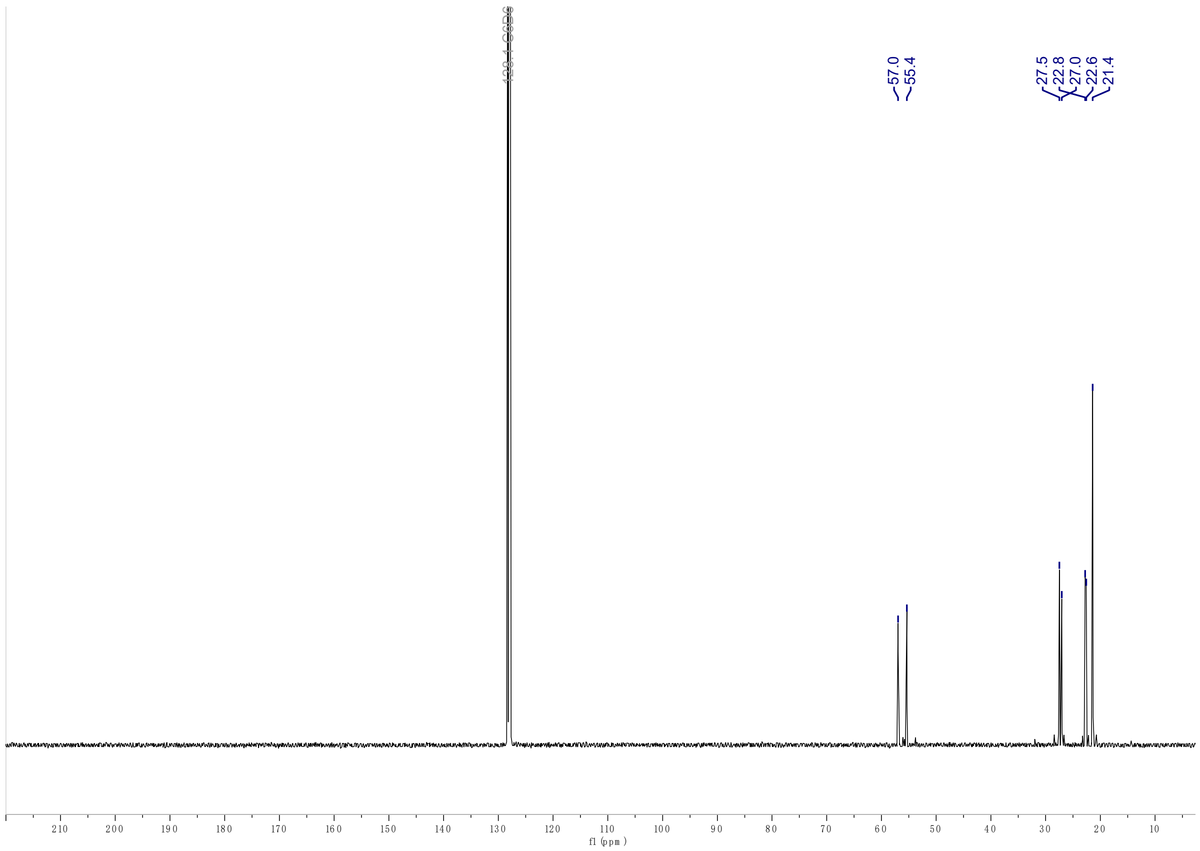

Figure S18. ^{13}C NMR (100.64 MHz, C_6D_6) of 2

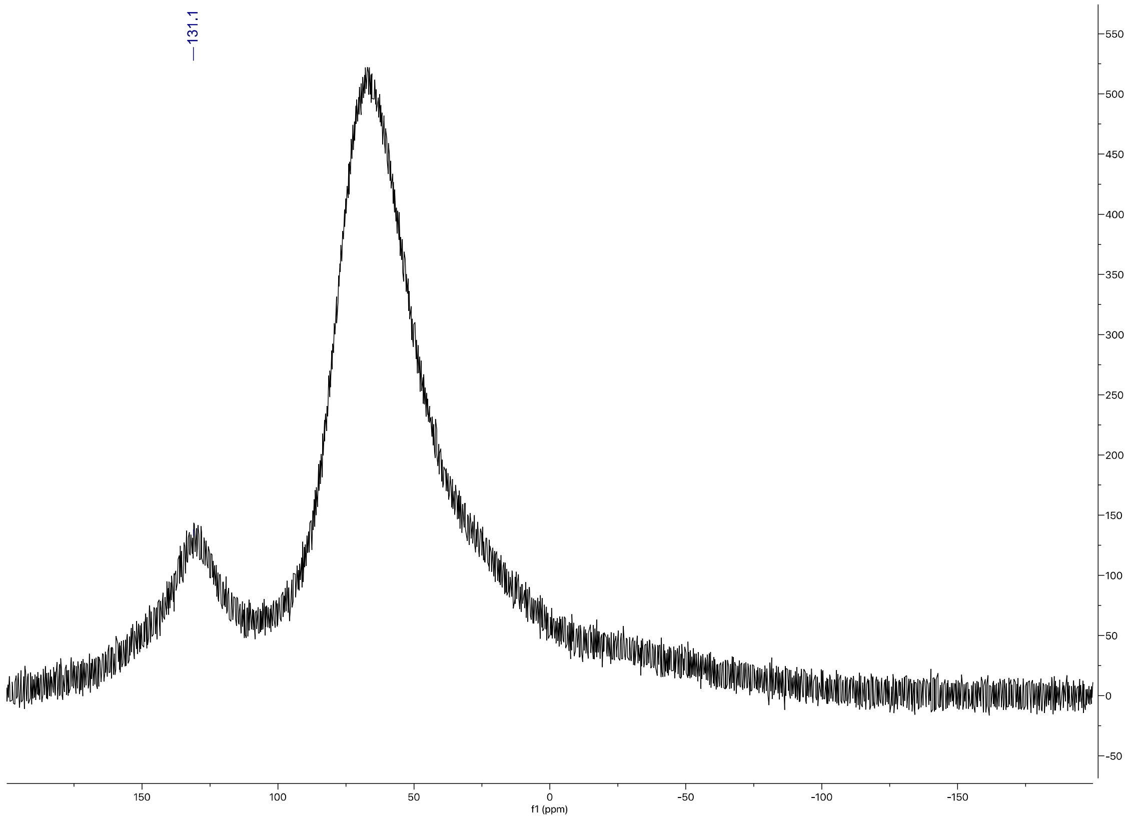

Figure S19. ^{27}Al NMR (104.27 MHz, C_6D_6) of **2**
